# Shape-changing chains for morphometric analysis of 2D and 3D, open or closed outlines

**DOI:** 10.1038/s41598-021-00911-5

**Published:** 2021-11-02

**Authors:** Bingjue Li, Shengmin Zhou, Andrew Peter Murray, Gérard Subsol

**Affiliations:** 1 School of Mechanical Engineering and Jiangsu Key Laboratory for Design and Manufacture of Micro/Nano Biomedical Instruments, School of Mechanical Engineering, Southeast University, Nanjing, 211189 Jiangsu China; 2 Design of Innovative Machines Lab, Department of Mechanical and Aerospace Engineering, University of Dayton, Dayton, OH 45469 USA; 3 ICAR Research-Team, Laboratory of Computer Science, Robotics and Microelectronics of Montpellier, University of Montpellier, CNRS, 34095 Montpellier, France

**Keywords:** Developmental biology, Ecology, Evolution, Plant sciences, Systems biology, Zoology, Ecology, Anatomy, Diseases, Signs and symptoms, Mechanical engineering

## Abstract

Morphometrics is a multivariate technique for shape analysis widely employed in biological, medical, and paleoanthropological applications. Commonly used morphometric methods require analyzing a huge amount of variables for problems involving a large number of specimens or complex shapes. Moreover, the analysis results are sometimes difficult to interpret and assess. This paper presents a methodology to synthesize a shape-changing chain for 2D or 3D curve fitting and to employ the chain parameters in stepwise discriminant analysis (DA). The shape-changing chain is comprised of three types of segments, including rigid segments that have fixed length and shape, scalable segments with a fixed shape, and extendible segments with constant curvature and torsion. Three examples are presented, including 2D mandible profiles of fossil hominin, 2D leaf outlines, and 3D suture curves on infant skulls. The results demonstrate that the shape-changing chain has several advantages over common morphometric methods. Specifically, it can be applied to a wide range of 2D or 3D profiles, including open or closed curves, and smooth or serrated curves. Additionally, the segmentation of profiles is a flexible and automatic protocol that can consider both biological and geometric features, the number of variables obtained from the fitting results for statistical analysis is modest, and the chain parameters that characterize the profiles can have physical meaning.

## Introduction

Morphometry is the quantitative analysis of form including shape and size. Morphometric analysis is widely adopted in biology, medical imaging, anthropology, and even fundamental science and engineering applications^1,2^. Based on the data being used, current morphometrics can be divided into traditional morphometrics and geometric morphometrics (GM). “Traditional” morphometric methods use multivariate statistical tools to analyze a small number of variables such as length measures and angles that characterize the overall form^3^. There are several difficulties for traditional morphometrics, such as determining the most appropriate size normalization method, identifying small changes that cannot be reflected by overall variables, and obtaining a graphical representation of the differences between shapes from the variables. “Geometric morphometrics”, including landmark-based methods and outline-based methods, has been the mainstream morphometric approach to study biological shape differences^4–7^. Geometric morphometrics can capture the complete geometric information of a morphological structure and retain the information intact throughout the analysis. Landmark-based methods compare the locations of landmarks or semilandmarks, the former of which are a set of anatomically homologous points, while the latter are interpolated points on smooth regions (curves or surfaces) that lack precise landmarks. For landmark-based methods, the biological homology of landmarks is always arguable, not to mention semilandmarks which are merely mathematically homologous. In addition, semilandmarks often requires comparing hundreds and thousands of points to achieve acceptable accuracy, thus data redundancy is also a problem for landmark-based methods^8^. Outline-based methods compare coefficients of mathematical functions used to fit points along the contours. Elliptic Fourier analysis (EFA) is one of the most popular approaches in outline-based methods^7,9,10^. EFA has several limitations too. First, EFA was not designed to match open curves. Second, it is susceptible to slight interference in the contour^11^. For shapes with complex boundaries, EFA requires a large number of harmonics to achieve satisfying fitting accuracy, and thus needs a comparison of numerous coefficients. These coefficients are mathematical variables and don’t have biological meaning. What is more, EFA produces troubling results when analyzing profiles which are prone to circular and/or bilateral symmetry^12^.

In this paper, we present a morphometric method to compare 2D or 3D outline curves (also called profiles), whether they are open or closed. This method is extended from the methodology for synthesizing planar shape-changing rigid-body mechanisms introduced by Murray et al.^13^. Such a mechanism performs a series of shape changes by altering its edge geometry which is formed by a chain of rigid bodies connected by revolute joints. Prismatic joints are also employed in the chain to extend the methodology for profiles of significantly different arc lengths^14^. In order to address growth factor of biological samples, a new type of segment called a G-segment is introduced^15^. So far, there are three types of segments in the chain. A mean segment, or M-segment, with fixed shape and size represents the average geometry of corresponding portions on profiles. A circular helical shaped segment or H-segment with constant curvature, constant torsion, and variable arc length, represents the average curvature and torsion of corresponding portions^16^). In planar cases, an H-segment has zero torsion and constant curvature, and is usually termed a C-segment (constant-curvature segment). A growth segment, or G-segment, with a fixed shape and variable size addresses the growth factor of biological samples. This method has been used to match several 2D and 3D biological profiles, including cochlea centerlines, sagittal skull profiles, transverse head circumferences, and lambdoid sutures^15,16^. However, further morphometric analysis based on the matching result was not performed in previous work.

The contribution of this paper are improvements to the methodology for fitting and analyzing curves in morphometry applications and realizing statistical analysis based on the chain parameters. The boundaries of anatomical structures can be too smooth to find definite homologous points (e.g., soft tissue like cochlea and corpus callosum) or so serrated that typically require numerous data for an accurate description (e.g., leaf and sagittal skull). Although the shape-changing chain method has been proven to fit such curves successfully, the key is to obtain a meaningful statistical result based on the chain parameters. Therefore, homology among profile curves must be addressed in the matching result. In this work, a number of segmentation points are determined according to biological knowledge and the geometrical features of each profile, dividing a profile into several sub-profiles. Next, each sub-profile is parted into one or more portions. The shape of each portion of the profile is approximated by a segment on the shape-changing chain. Compared with typical mechanical design problems which usually have a few target profiles, the number of biological samples easily reaches several dozens and sometimes hundreds. Hence, the synthesis methodology needs a more efficient optimization strategy for such a large number of profiles. An iterative optimization process targeting poorly fitted profiles at each step is proposed. After the optimal shape-changing chain is obtained, chain parameters such as the relative angles between segments and the length ratios of C-segments (or H-segments) and G-segments are studied using stepwise discriminant analysis (DA). Examples include the lateral shapes of fossil hominid mandibles (94 specimens, 4 groups) from the work of^17^, leaf contours (145 specimens, 9 groups), and 3D suture curves on human skulls (63 specimens, 4 groups) from a coronal craniosynostosis study^18,19^. Results show that the presented method is capable of fitting 2D or 3D, open or closed, smooth or serrated curves, and achieve a satisfying classification accuracy based on a moderate number of parameters. In addition, the fitting chain provides a physical interpretation of the shape differences between samples. Moreover, a single technique is provided that handles all morphometric challenges with accepting high accuracy.

## Method

### Review of the segmentation procedure

**Figure 1:**
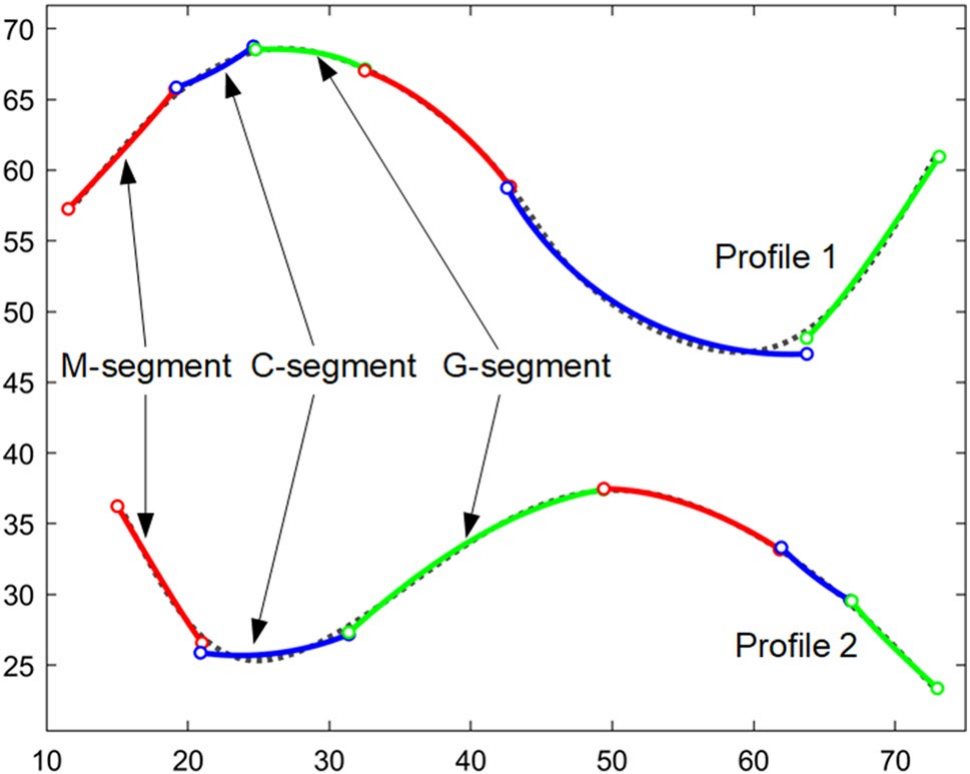
Two 2D profiles are matched by a single shape-changing chain.

This section provides a brief review of the general procedure of synthesizing a shape-changing chain to match a set of profiles. More details can be found in^20,21^ and^16^. The process starts with assigning a set of design profiles that represent the biological forms to be studied. Design profiles can be open or closed curves of different arc lengths, i.e. the shape-changing chain can match curves of their original size without scaling (normalization) in advance. Design profiles are then converted into target profiles which are piecewise linear curves with roughly equal piece lengths. Thus, the arc length of a design profile is quantified by the number of linear pieces in its corresponding target profile. The total number of points on the ${j}{\text{th}}$ target profile is denoted as ${N}_{j}$. In following procedures, target profiles are analyzed and referred to as profiles for short. In the segmentation process, target profiles are divided into several portions. Each portion is approximated by a segment of the same number of pieces in the shape-changing chain. To do so, a segment type vector **V** that specifies the number, type, and order of segments is assigned manually. A *p* × *q* segment matrix $\mathbf{S}\mathbf{M}$ is then generated to designate the number of pieces in each portion/segment for each profile, where *p* is the number of profiles and *q* is the number of portions/segments. For example, the target profiles shown in Fig. 1 contain 1131 and 1001 pieces respectively. The segment type vector and the segment matrix are

$$\mathbf{V}=\left[\begin{array}{cc}\begin{array}{ccc}{\text{M}}& {\text{C}}& {\text{G}}\end{array}& \begin{array}{ccc}{\text{M}}& {\text{C}}& {\text{G}}\end{array}\end{array}\right],$$

$$\mathbf{S}\mathbf{M}{\left[{m}_{j}^{e}\right]}_{2\times 6}=\left[\begin{array}{cc}\begin{array}{ccc}{m}_{1}^{1}& {m}_{1}^{2}& {m}_{1}^{3}\\ {m}_{2}^{1}& {m}_{2}^{2}& {m}_{2}^{3}\end{array}& \begin{array}{ccc}{m}_{1}^{4}& {m}_{1}^{5}& {m}_{1}^{6}\\ {m}_{2}^{4}& {m}_{2}^{5}& {m}_{2}^{6}\end{array}\end{array}\right]=\left[\begin{array}{cc}\begin{array}{ccc}207& 90& 507\\ 207& 115& 213\end{array}& \begin{array}{ccc}130& 98& 99\\ 130& 127& 209\end{array}\end{array}\right].$$

Note that in the segment matrix, columns corresponding to M-segments have equal numbers of pieces, and the sum of each row is the total number of pieces in each target profile. A set of points called segmentation points divide a profile into portions. The location of the ${e}{\text{th}}$ segmentation point on the ${j}{\text{th}}$ profile is given as

1 $${k}_{j}^{e}=\left\{\begin{array}{l}1 e=1,\\ 1+\sum_{i=1}^{e-1}{m}_{j}^{i} 2\le e\le q,\\ {N}_{j} e=q+1.\end{array}\right.$$

In Eq. (1), the starting point and the endpoint on a profile are considered as the first and the last segmentation points on that profile. A *p* × *q* error matrix $\mathbf{E}\mathbf{M}$ evaluates the matching error of each segment for each profile. Entry ${E}_{j}^{e}$ is calculated as the maximum point-to-point matching error of the ${e}{\text{th}}$ segment compared to the ${j}{\text{th}}$ profile,

2 $${E}_{j}^{e}=\underset{i=1,\dots ,{m}_{j}^{e}+1}{\text{max}}\Vert {\overline{\mathbf{z}} }_{{j}_{i}}^{e}-{\mathbf{z}}_{{j}_{i+{k}_{j}^{e}-1}}\Vert ,$$

where ${\overline{\mathbf{z}} }_{{j}_{i}}^{e}$ is the coordinates of the ${i}{\text{th}}$ point on the ${e}{\text{th}}$ segment, ${\mathbf{z}}_{{j}_{i+{k}_{j}^{e}-1}}$ is the coordinates of the point corresponding to ${\overline{\mathbf{z}} }_{{j}_{i}}^{e}$ on the ${j}{\text{th}}$ profile. For the chain shown in Fig. 1, the error matrix is

$$\mathbf{E}\mathbf{M}={\left[{E}_{j}^{e}\right]}_{2\times 6}=\left[\begin{array}{cc}\begin{array}{ccc}{E}_{1}^{1}& {E}_{1}^{2}& {E}_{1}^{3}\\ {E}_{2}^{1}& {E}_{2}^{2}& {E}_{2}^{3}\end{array}& \begin{array}{ccc}{E}_{1}^{4}& {E}_{1}^{5}& {E}_{1}^{6}\\ {E}_{2}^{4}& {E}_{2}^{5}& {E}_{2}^{6}\end{array}\end{array}\right]$$

$$=\left[\begin{array}{cc}\begin{array}{ccc}0.5750& 0.5599& 1.1759\end{array}& \begin{array}{ccc}0.8270& 0.2809& 0.1215\end{array}\\ \begin{array}{ccc}0.5750& 0.7843& 0.4901\end{array}& \begin{array}{ccc}0.8270& 0.3837& 0.2854\end{array}\end{array}\right]$$

Besides $\mathbf{E}\mathbf{M}$, a number of error metrics are also used for evaluating the matching error and optimization (see Table 1). These error metrics provides a more comprehensive evaluation of the matching results.

**Table 1:** Metrics for evaluating matching error.

Error metrics	Formula	
Maximum point-to-point error of all segments compares to all profiles	$${E}_{\text{max}}=\underset{\begin{array}{c}e=1,\dots ,q\\ j=1,\dots ,p\end{array}}{\text{max}}\left({E}_{j}^{e}\right)$$	(3)
Average value of the error matrix $\bf EM$	$\overline{E }=\frac{1}{pq}\sum_{e=1}^{q}\sum_{j=1}^{p}{E}_{j}^{e}$	(4)
Average value of the maximum point-to-point error of all the segments compared to the ${j}{\text{th}}$ profile	${\overline{E} }_{j}=\frac{1}{q}\sum_{e=1}^{q}{E}_{j}^{e}$	(5)
Average point-to-point error of all segments on the shape-changing chain compared to the ${j}{\text{th}}$ profile	${\tilde{E }}_{j}=\frac{1}{{N}_{j}}\sum_{e=1}^{q}\sum_{i=1}^{{m}_{j}^{e}+1}\Vert {\overline{\mathbf{z}} }_{{j}_{i}}^{e}-{\mathbf{z}}_{{j}_{i+{k}_{j}^{e}-1}}\Vert$	(6)

The segment matrix is optimized according to the error matrix $\mathbf{E}\mathbf{M}$: Segments of lower error are lengthened, segments of higher error are shortened, and the total number of pieces in all segments remains the same for each profile. This process is repeated iteratively until an optimal $\mathbf{S}\mathbf{M}$ is obtained^20^. At this step, segments are aligned with their corresponding portions on each profile. For mechanical design applications, segments need to be connected by revolute joints or fused at a fixed angle to form a continuous chain. Note that connection is not necessary for morphometric applications, as the angle of a revolute joint (representing the rotation of a region) can be measured as the difference of the orientations of two segments. In addition, eliminating the connection avoids enlarging matching error and reduces computational work.

### Types of segments

**Figure 2:**
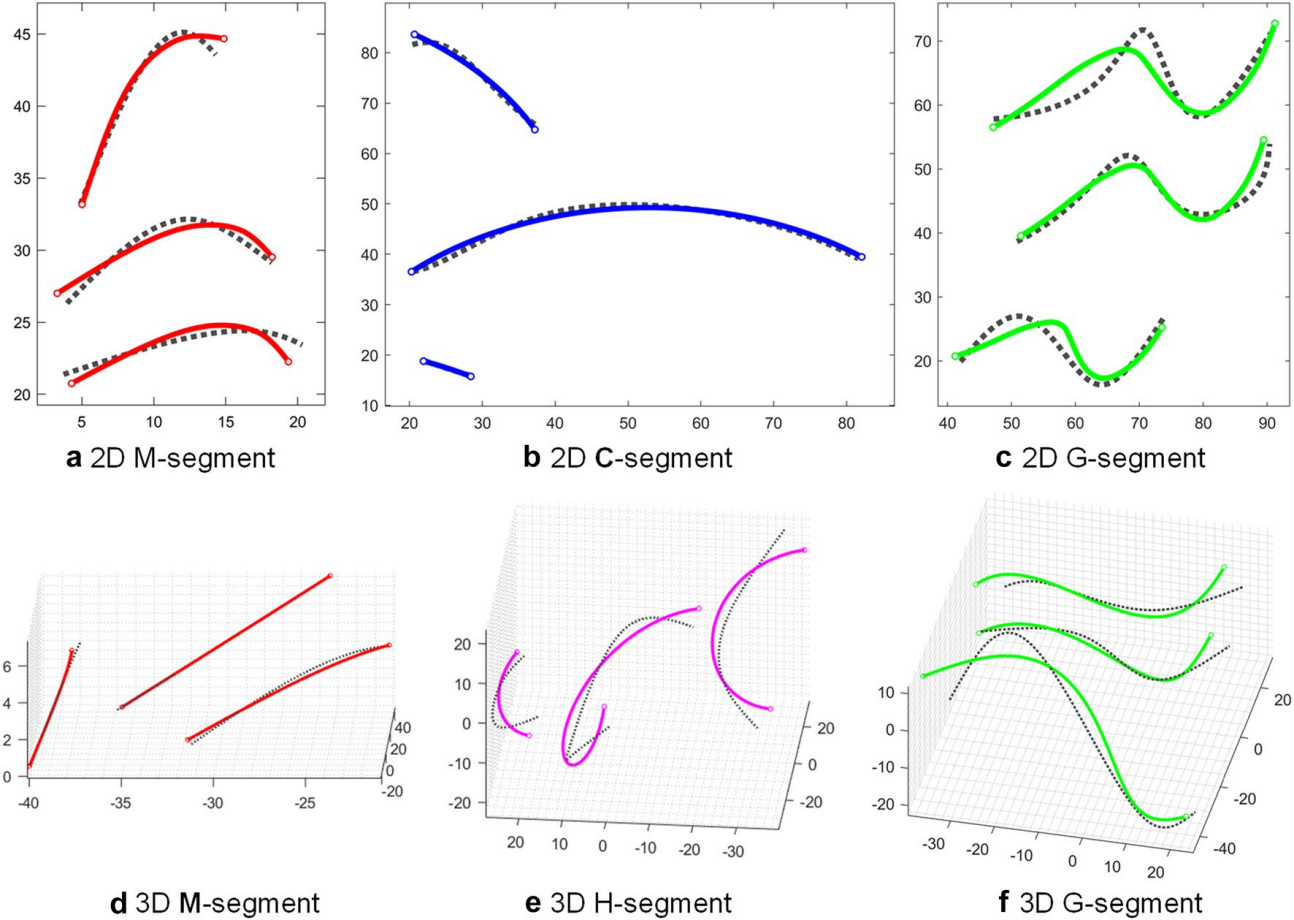
Different types of segments (shown in solid lines) approximate portions of 2D (first row) and 3D (second row) target profiles (shown in dashed lines).

There are three types of segments: M-segments, H-segments (whose special case is the C-segment), and G-segments. An illustration of these segments is shown in Fig. 2. A discussion about their geometric features now follows.

An M-segment is akin to a rigid body that approximates portions that contain the same number of pieces and represents their average form (including shape and size). Therefore, M-segments are used to represent regions of the samples exhibiting small variations. Since M-segments don’t introduce additional variables (degrees of freedom), they are favorable in composing the chain for mechanical design and morphometric analysis.

An H-segment is a circular helical shape that has the average curvature and torsion of the corresponding portions on 3D target profiles. In planar cases, the torsion of an H-segment is zero and a planar H-segment is a circular arc called a C-segment. An H-segment or a C-segment can be mechanically implemented by connecting two or more helical or arc-shaped links with a prismatic joint. They were introduced to approximate profiles of significantly different arc lengths for shape-changing mechanism design applications such as morphing wings^22^ or variable-geometry dies^23^. If the curvature is a non-zero value, then the orientation of a linear piece on the H- or C-segment changes with the arc length. Therefore, H- and C-segments can be used to model growth as well as rotation of a region. Since H- and C-segments have constant curvature and torsion, they are not suitable to match portions that have large changes in curvature and torsion, especially those that contain sharp corners^21^. In the statistical analysis that follows, their arc lengths, or more conveniently the numbers of pieces contained in corresponding portions at different profiles, can be compared. Assume the *e*th segment is an H-segment or C-segment, the corresponding portion on the *j*th profile contains ${m}_{j}^{e}$ pieces. Among all profiles, the *r*th profile contains the most pieces in the *e*th portion. Then, the ratio of number of pieces contained in the *e*th portion on the *j*th profile is calculated as

7 $${g}_{j}^{e}=\frac{{m}_{j}^{e}}{{m}_{r}^{e}}.$$

G-segments can replace H- or C-segments to address the growth factor between specimens for morphometric applications^15^. A G-segment is resizable and represents the average shape of portions that contain different numbers of pieces, thus G-segments usually have better shape-matching ability than H- and C-segments. Similarly, the numbers of pieces contained in a G-segment at different profiles are compared using Eq. (7) in the statistical analysis that follows.

### Improvements

#### Segmentation techniques

The shape-changing chain method has been successfully employed to match 2D or 3D, open or closed, smooth or serrated profiles. Examples include transverse head growth of children, sagittal skull evolution of human, 2D and 3D human cochlea centerlines, and 3D suture curves on human skulls^16,21^. However, a good matching result doesn’t always yield a meaningful interpretation or accurate grouping result in morphometric analysis. The key is to obtain homologous segmentation points. Sometimes, homologous points can be determined with biological knowledge of the samples. For example, the anatomical intersection point of the sagittal curve and the lambdoid suture on a human skull can be used as a segmentation point for these two curves. Other times, geometric features can help to locate homologous points. Although it is arguable to equate mathematical correspondence with biological homology, their differences are usually very small and do not significantly affect the final result. Therefore, a method is proposed to determine segmentation points based on geometric properties of target profiles. This method divides a target profile into several sub-profiles at convex or concave points, and then further divides sub-profiles into portions to be approximated by segments.

Let ${\mathbf{z}}_{{j}_{i}}$ denote the coordinates of the ${i}{\text{th}}$ point on the ${j}{\text{th}}$ target profile, then the ${i}{\text{th}}$ piece is a vector formed by the ${i}{\text{th}}$ point and the ${(i+1)}{\text{th}}$ point as ${\mathbf{z}}_{{j}_{i+1}}-{\mathbf{z}}_{{j}_{i}}$. The relative angle between the ${(i-1)}{\text{th}}$ piece and ${i}{\text{th}}$ piece on the ${j}{\text{th}}$ profile, ${\beta }_{{j}_{i}}^{1}$, is determined by three neighboring points ${\mathbf{z}}_{{j}_{i-1}}$, ${\mathbf{z}}_{{j}_{i}}$, and ${\mathbf{z}}_{{j}_{i+1}}$ as

8 $${\beta }_{{j}_{i}}^{1}={\text{cos}}^{-1}\left(\frac{\left({\mathbf{z}}_{{j}_{i+1}}-{\mathbf{z}}_{{j}_{i}}\right)\cdot \left({\mathbf{z}}_{{j}_{i}}-{\mathbf{z}}_{{j}_{i-1}}\right)}{\Vert {\mathbf{z}}_{{j}_{i+1}}-{\mathbf{z}}_{{j}_{i}}\Vert \cdot \Vert {\mathbf{z}}_{{j}_{i}}-{\mathbf{z}}_{{j}_{i-1}}\Vert }\right).$$

An angle threshold $T$ is set to capture convex or concave points with high relative angle. Since Eq. (8) calculates the relative angle between two neighboring pieces, small sharp burrs can also be recognized. Therefore, Eq. (8) can’t exclude local noise from globally sharp corners, which is troublesome for profiles that have serrated edges. To solve this problem, the interval between the three points to calculate the relative angle is increased, thus the relative angle calculated at point ${\mathbf{z}}_{{j}_{i}}$ becomes

9 $${\beta }_{{j}_{i}}^{k}={\text{cos}}^{-1}\left(\frac{\left({\mathbf{z}}_{{j}_{i+k}}-{\mathbf{z}}_{{j}_{i}}\right)\cdot \left({\mathbf{z}}_{{j}_{i}}-{\mathbf{z}}_{{j}_{i-k}}\right)}{\Vert {\mathbf{z}}_{{j}_{i+k}}-{\mathbf{z}}_{{j}_{i}}\Vert \cdot \Vert {\mathbf{z}}_{{j}_{i}}-{\mathbf{z}}_{{j}_{i-k}}\Vert }\right)$$

**Figure 3:**
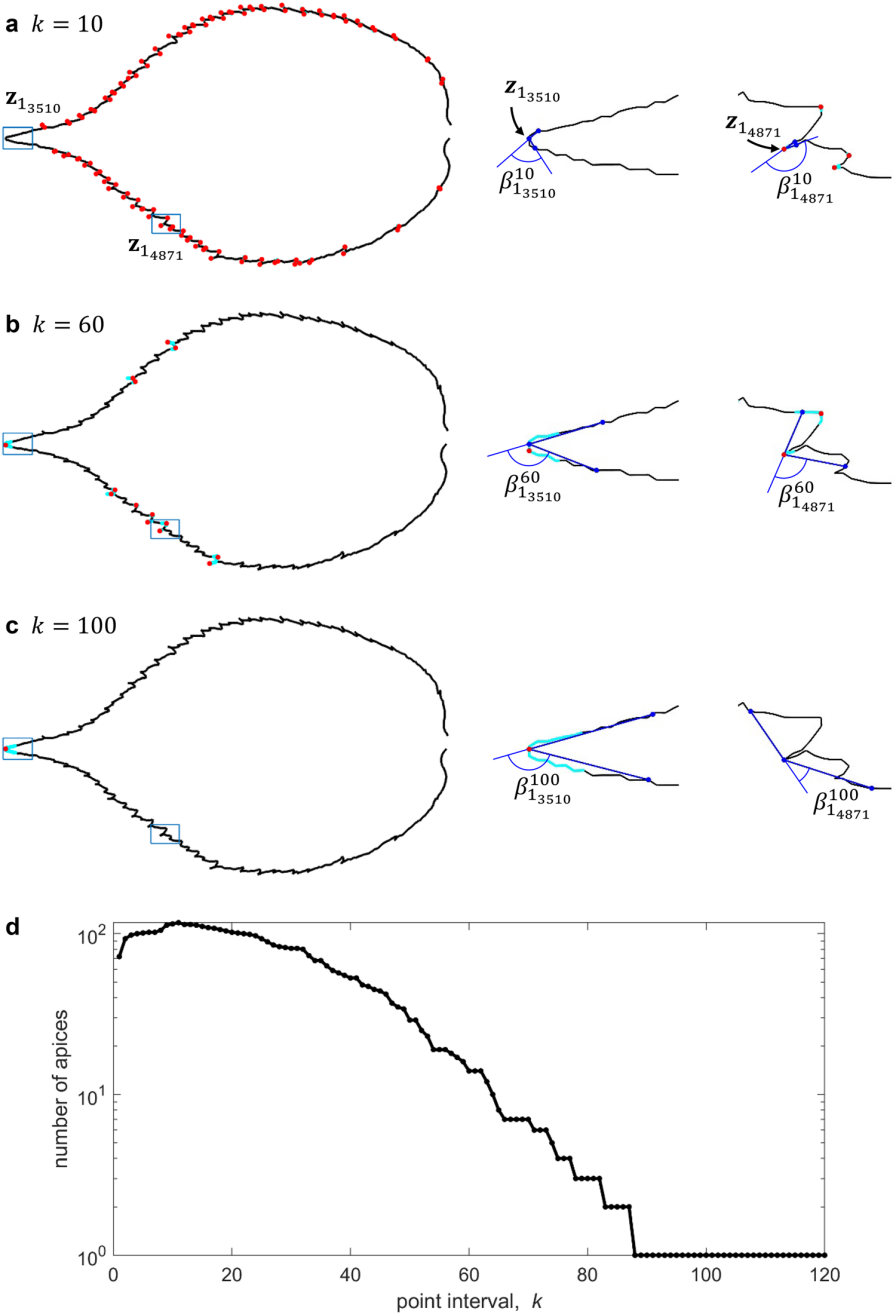
The process of locating a globally sharp corner (the tip) on the contour of a *cherry* leaf ($j=1$ from a total of 145 leaf profiles). The angle threshold $T$ is set to 90°. (**a**–**c**) The apices detected via the relative angle at different point intervals. Cyan portions are where the relative angle is above $T$, and red points are the point where the largest relative angle occurs within that portion. The middle column and the right column shows the relative angle calculated at point ${{\varvec{z}}}_{{1}_{3510}}$ (the tip) and at point ${{\varvec{z}}}_{{1}_{4871}}$ (local burr) respectively. (**d**) The number of apices identified on the leaf contour while the point interval $k$ varies.

By adjusting the point interval $k$ in Eq. (9) and selecting a proper angle threshold $T$, most local burrs can be filtered out, while global apices or convex/concave points in a smooth region can be located and used as segmentation points to divide a target profile into sub-profiles. Figure 3 shows the procedure of identifying the apex (i.e. the tip) of a *cherry* leaf profile. The *cherry* leaf profile contains many burrs which are not desired to be identified as sharp corners. Observe that the relative angle of a local burr, such as point ${\mathbf{z}}_{{1}_{4871}}$, is high with a small interval $k$ and reduces as $k$ expands, while a global apex such as point ${\mathbf{z}}_{{1}_{3510}}$ remains a high relative angle with a sufficient interval $k$. Therefore, the number of apices detected on the leaf profile generally declines as the point interval increases (when $k\ge 11$). With sufficient interval, the number of apices remains at 1, which means all local burrs are filtered out and only the tip of the leaf is identified as the sharp corner.

**Figure 4:**
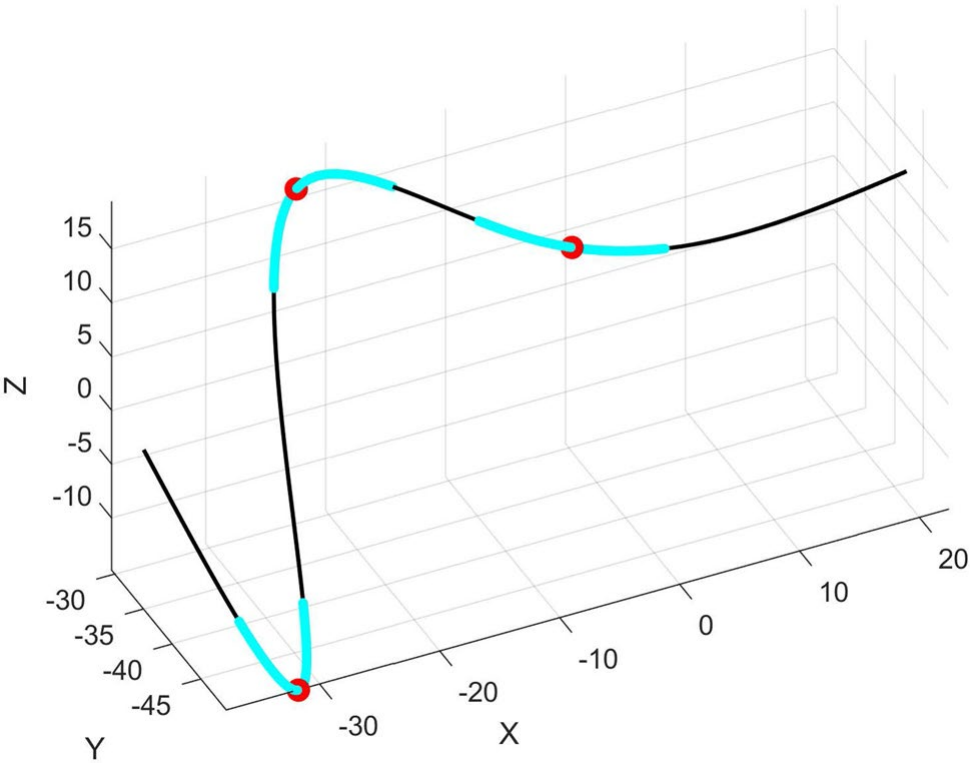
Detecting concave/convex points (shown in red) on a smooth 3D curve using relative angle. Cyan portions are where the relative angle is above $T$.

Using this method, an equal or similar number of global apices can be located on leaves that belong to the same genre. These apices are similar to Type 2 landmarks in GM analysis and herein are used as segmentation points. As shown in Fig. 4, this strategy can also be applied to smooth curves without obvious sharp corners to locate concave or convex points with higher curvature (and torsion) as segmentation points. This method brings two benefits. First, it reduces matching error by avoiding a segment being arranged across high-curvature regions on a profile. Second, profiles are divided at points that have correspondence rather than random segmentation points.

Note that segmentation points determined using this method may need further selection or supplementation to make profiles that belong to the same group have a consistent number of homologous points. After this step, all profiles are divided into a same number of sub-profiles. Next, more segmentation points can be generated within each sub-profile to increase matching accuracy. In this work, the location of segmentation points within a sub-profile is initial randomly determined and then subject to optimization.

For very smooth profiles, such as the centerline of cochlea, locating accurate biological or mathematical homologous points is difficult. These types of profiles can be fitted by a shape-changing chain with low matching error, but it is still challenging to obtain a rational interpretation of the fitting results. Braga et.al employed elastic curve theory based on Riemann differential geometry to analyze the sex-typed shape difference of cochlea^24^. Sliding semilandmarks is another method used to deal with smooth curves^25,26^. Note that the homology in mathematics is not the same as biological homology, and the semilandmark method is a mathematical tool that figures out homologous points. As a result, obtaining homologous segmentation points in mathematical concepts should be proposed for more precise fitting and analysis in the future.

#### Optimization

After segmentation points are randomly generated for each sub-profile, their locations can be further optimized. In previous work, a piece-by-piece optimization according to the error matrix is performed on all profiles iteratively^20^. This optimization method, however, is very time consuming when dealing with dozens or hundreds of profiles. On the other hand, generating random locations of the segmentation points on a profile is much more rapid. Therefore, an optimization process which modifies the lengths of segments for only a number of worst matched profiles is proposed.

The optimization process starts with calculating the average point-to-point matching error ${\tilde{E }}_{j}$ between the shape-changing chain and each profile using Eq. (6). Next, all profiles are sorted in the order of their matching error. A number of profiles that have the highest matching error would be re-segmented, while the rest remain unchanged. Note that since the length of an M-segment is consistent among all profiles, so for the profiles to be optimized, only the lengths of G-segments and H-segments (or C-segments) are regenerated. This process is repeated until the matching error stops decreasing.

## Morphometric examples and results

### 2D mandible outlines

**Figure 5:**
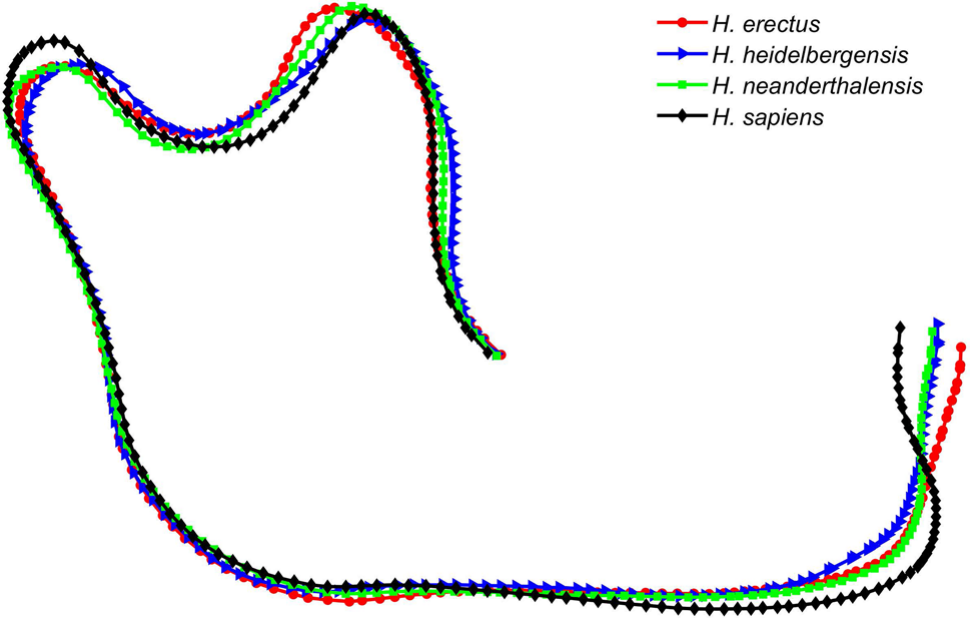
Mean mandibular shapes of samples from *H. erectus* (red circles), *H. heidelbergensis* (blue triangles), *H. neanderthalensis* (green squares), and *H. sapiens* (black diamonds).

In^17^, elliptical Fourier analysis (EFA) is employed to investigate the lateral shape difference between 106 fossil mandibles of 5 groups: *A. robustus* ($n=7$), *H. erectus* ($n=12$), *H. heidelbergensis* ($n=4$), *H. neanderthalensis* ($n=22$), and *H. sapiens* ($n=61$). In the presented work, the authors apply the shape-changing chain method to the same dataset. Twelve samples were suppressed, including all 7 *A. robustus* samples, 4 *H. neanderthalensis* samples, and one *H. sapiens* sample. Therefore, 94 mandible profiles of 4 groups of the ancient human are analyzed: *H. erectus* ($n=12$), *H. heidelbergensis* ($n=4$), *H. neanderthalensis* ($n=18$), and *H. sapiens* ($n=60$). The dataset is in the form of Cartesian coordinates of points along the mandible boundary. Note that the shape-changing chain method does not require pre-alignment of curves or removal of the size factor. However, the mandible profile dataset the authors obtained had already lost the information of the original sample sizes. Therefore, only normalized mandibular shapes are compared herein. Figure 5 illustrates the mean shape of each group of profiles by aligning all profiles in the set using a standard Procrustes superimposition (PS) which includes translation, scaling, and rotation of the profiles^27^.

**Figure 6:**
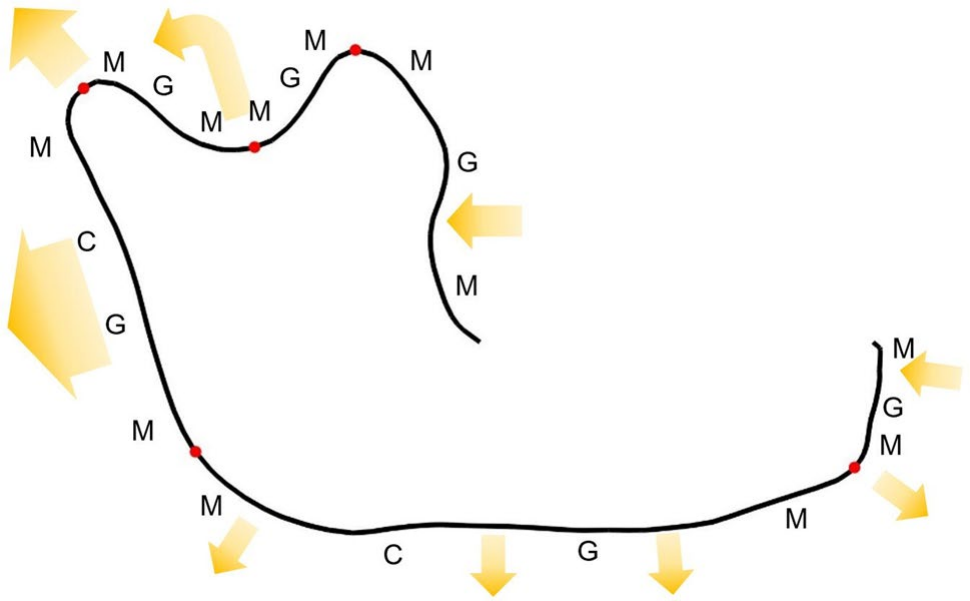
A profile ($j=1$) from the *H. erectus* group: Five apices (red circle) are located using the relative angle method ($k=150$, $T=20^\circ$) and divide the profile into six sub-profiles. The arrows represent the growth pattern of the mandible^28^.

Using the relative angle method ($k=150$, $T=20^\circ$), five apices are reserved, constituting six sub-profiles for each profile. An illustration of the location of the apices on a mandible profile is shown in Fig. 6. Each sub-profile is then matched with a shape-changing chain individually. The determination of segment type vector of each sub-profile refers to the growth mechanism of mandible proposed by Enlow et al.^28^. As shown in Fig. 6, the mechanism of mandible growth involves bone resorption (indicated by the arrows pointing towards the mandible contour) and bone deposition (indicated by the arrows pointing out of the mandible contour). Although the whole mandible's displacement direction is forwards and downwards, the reconstruction of the ascending limb is generally backwards and upwards. G-segments and C-segments are employed to approximate the growing portions in target profiles and characterize the difference in profile lengths.

**Figure 7:**
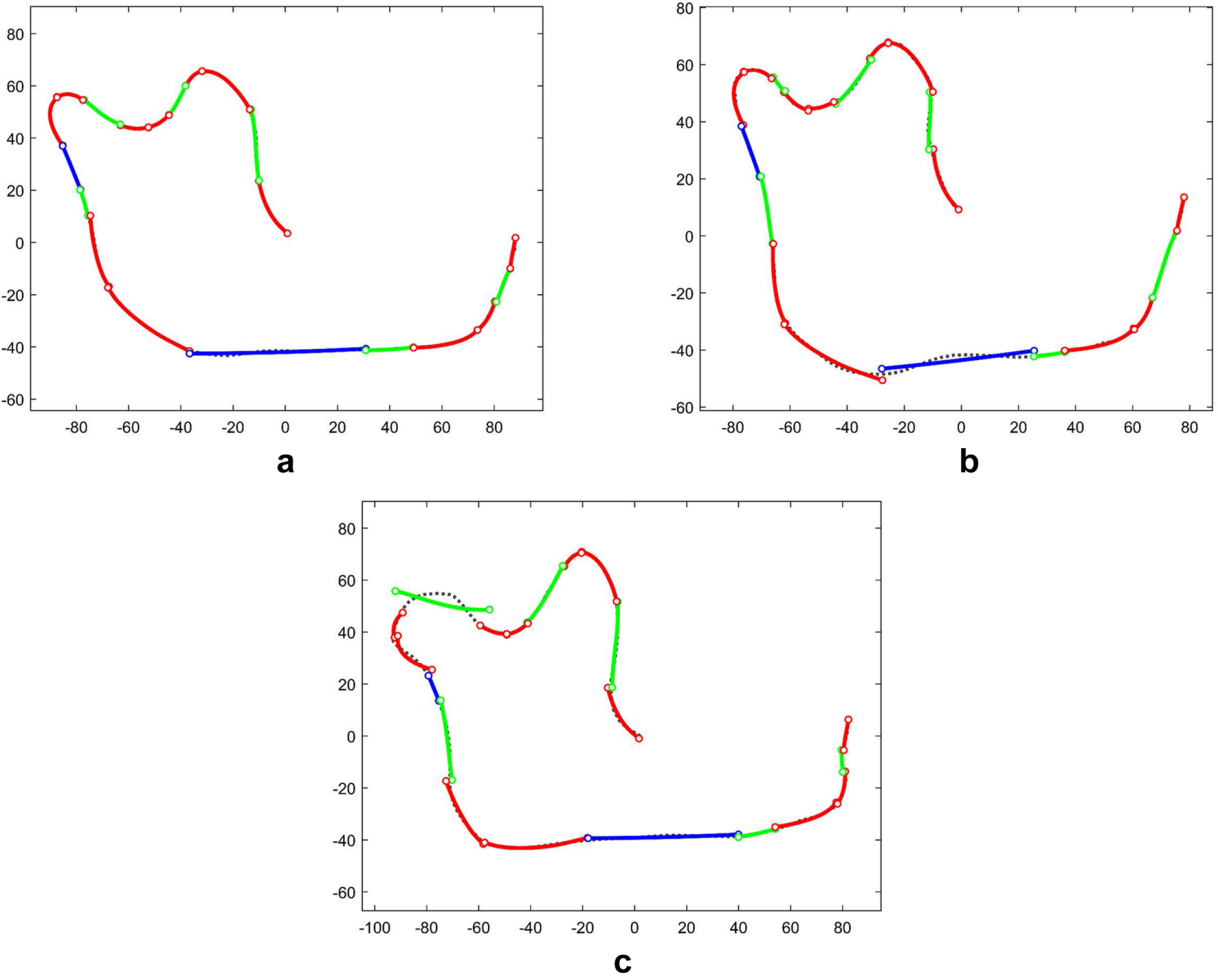
The fitting result of 94 human mandibles. (**a**) The best match (the 4th profile—*H. erectus*, ${\tilde{E }}_{4}=0.3924$); (**b**) The match with error closest to $\overline{E }$ (the 6th profile—*H. erectus*, ${\tilde{E }}_{6}=0.6006$); (**c**) The worst match (the 13th profile—*H. heidelbergenis*, ${\tilde{E }}_{13}=1.0771$).

Note that the growths of the inferior edge of the mandibular body and the posterior edge of the mandibular ramus are more significant than the rest parts of the mandible profile. The 94 mandible profiles are then matched with a shape-changing chains using the following scheme. The segment vectors for the first, second, third, and sixth sub-profiles are defined as $\left[{\text{MGM}}\right]$ alike, and the segment vectors for the fourth and fifth sub-profiles are both defined as $\left[{\text{MCGM}}\right]$, where the C-segments and G-segments are used to capture the difference in arc lengths. Therefore, the overall segment vector is

$$\mathbf{V}=\left[\text{M G M M G M M G M M C G M M C G M M G M} \, \right]\text{,}$$

where there are a total of 20 segments—12 M-segments, 2 C-segments, and 6 G-segments. After the segment type vector is defined, the shape-changing chain is generated to match the target mandible profiles and then is optimized for each sub-profile. The maximum and mean error of all profiles of the final matching result are ${E}_{\text{max}}=8.0863$ and $\overline{E }=0.6009$ units, respectively. Figure 7 shows the best (a), the average (b), and the worst match (c) according to ${\tilde{E }}_{j}$. Note that in the worst match, the G-segment at the condyle (head) of the mandible causes the largest matching error. This is because the third primary segmentation point (between the two M-segments that follow) identified using the relative angle method for this specific profile is not at the tip of the condyle as the majority of the profiles.

The orientation difference between two neighboring segments reflects the rotational angle between them, and thus are employed in the statistical analysis in the next step. Denote the direction of a vector $\mathbf{u}={\left\{{u}_{x},{u}_{y}\right\}}^{T}$ as $\angle \left(\mathbf{u}\right)$, then the orientation change between the ${e}{\text{th}}$ and the ${(e+1)}{\text{th}}$ segments on the ${j}{\text{th}}$ profile is calculated as the difference between the direction of the last piece on the ${e}{\text{th}}$ segment and the direction of the first piece on the ${(e+1)}{\text{th}}$ segment

10 $${\sigma }_{j}^{e}=\angle \left({\overline{\mathbf{z}} }_{{j}_{2}}^{e+1}-{\overline{\mathbf{z}} }_{{j}_{1}}^{e+1}\right)-\angle \left({\overline{\mathbf{z}} }_{{j}_{{m}_{j}^{e}+1}}^{e}-{\overline{\mathbf{z}} }_{{j}_{{m}_{j}^{e}}}^{e}\right), \forall e=1,\dots ,q-1 j=1,\dots ,p.$$

**Figure 8:**
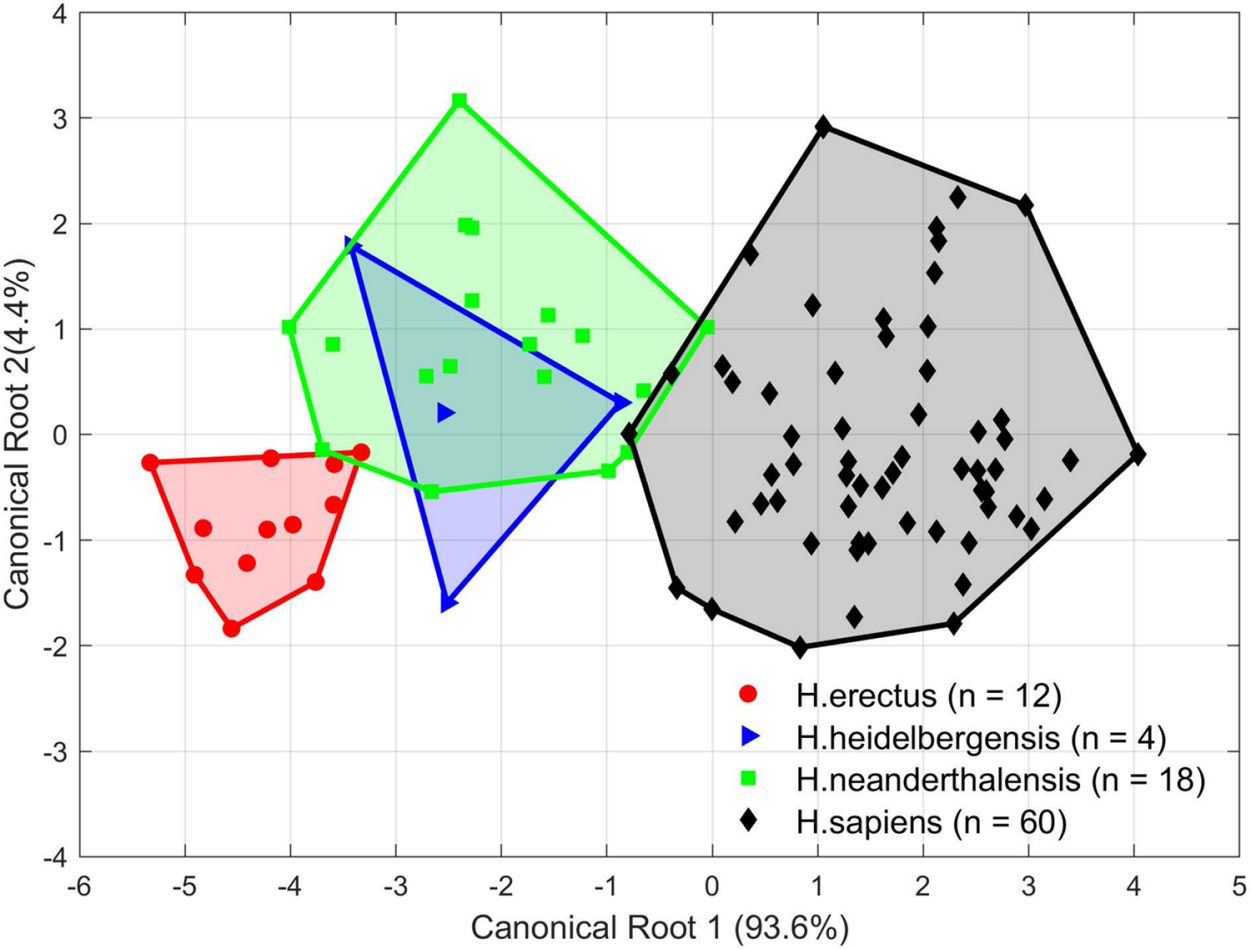
Canonical plot of the 94 human mandibles from four groups (*H. erectus*, *H. heidelbergensis*, *H. neanderthalensis*, and *H. sapiens*) based on the orientation changes between segments (19 variables).

In the mandible example, 19 angular variables are generated from 20 segments. As in^17^, a stepwise discrimination analysis (DA) is conducted (in IBM SPSS 22) to figure out the relationship among the four homo groups. DA is a supervised classification method and returns $g-1$ canonical components among $g$ groups of samples^29^. Figure 8 shows the convex hull of four homo genus plotted with the first and the second canonical components. The three main groups: *H. erectus*, *H. neanderthalensis*, and *H. sapiens*, are separated from each other in the direction of the first canonical component. *H. heidelbergensis* and *H. neanderthalensis* have an overlap in the direction of the second canonical component. In stepwise DA, leave-one-out cross-validation (LOOCV) is applied to verify the stability of the linear model. As a result, the prediction accuracy is 91.5% and the cross-validation accuracy is 80.9%. This DA result suggests that the shape-changing chain method is useful in analyzing 2D shapes. The classification matrices of original prediction and LOOCV are presented in Table 2, showing the details of discrimination of the four mandibular shape groups.

**Table 2:** Classification matrices of the original DA and cross-validated prediction of 94 human mandibles.

Group	1		2		3		4		Totals	
	Count	Percentage	Count	Percentage	Count	Percentage	Count	Percentage	Count	Percentage
**Original**										
1. *H. erectus*	11	**91.7**	1	8.3	0	0.0	0	0.0	12	100.0
2. *H .heidelbergensis*	0	0.0	3	**75.0**	1	25.0	0	0.0	4	100.0
3. *H. neanderthalensis*	1	5.6	2	11.1	14	**77.8**	1	5.6	18	100.0
4. *H. sapiens*	0	0.0	2	3.3	0	0.0	58	**96.7**	60	100.0
**Cross-validated**										
1. *H. erectus*	10	**83.3**	2	16.7	0	0.0	0	0.0	12	100.0
2. *H .heidelbergensis*	1	25.0	0	0.0	3	75.0	0	0.0	4	100.0
3. *H. neanderthalensis*	3	16.7	4	22.2	10	**55.6**	1	5.6	18	100.0
4. *H. sapiens*	0	0.0	2	3.3	2	3.3	56	**93.3**	60	100.0

Note that the classification results as shown in Fig. 8 and Table 2 are in accordance with the results obtained with EFA in^17^. The high misclassification rate of *H. heidelbergensis* and its distribution on the canonical plot are also in keep with the mainstream opinion that *H. heidelbergensis* is a chronospecies evolving from *H. erectus* and is considered as the most recent common ancestor (MRCA) between *H. sapiens* and *H. neanderthalensis*. In the work of Lestrel et al. based on EFA, 20 harmonics are employed to match 106 mandibular shapes, producing 82 Fourier descriptors^17^. Then, 12 distances from the centroid to specified points on each mandible’s contour are used in statistical analysis. Compared to their study, the shape-changing chain method generates only a total of 28 variables (20 orientations of all segments and 8 arc lengths of C-segments and G-segments). The differences of orientations between neighboring segments is then calculated and generates 19 variables to be analyzed in stepwise DA. Table 3 shows a comparison of the variables generated in the approximation of curves and used for statistical analysis with the shape-changing chain method and EFA. The shape-changing chain method performs a satisfying approximation result of the mandibular shapes with much fewer variables compared with EFA.

**Table 3:** Numbers of variables used in the shape-changing chain method and in EFA^17^ for fitting and analyzing human mandible profiles.

Method	Variables in fitting curves	Variables to be analyzed	Variables totally obtained	Prediction accuracy	Cross-validation accuracy
Shape-changing chain method	28	19	28	91.5%	80.9%
EFA	82	12	94	91.5%	Not provided

### 2D leaf outlines

Leaf classification is a typical problem that has been studied with various methods, such as artificial neural networks (ANN)^30^, image moments^31^, and EFA^9^. In addition, many leaves have a symmetrical shape creating issues for effective EFA^12^. Using the shape-changing chain method, the fitting result reveals the growth of portions on the contour and the rotation between them. This kind of information can be used in statistical analysis. Although other methods which also make use of non-shape information (size, color, etc.) have been very convenient and efficient in recognizing leaf genera, leaf matching and classification remains a problem to test the ability of the shape-changing chain method to fit and compare profiles with complicated and largely varying shapes. In this example, nine groups of 145 leaves are studied (see the groups and the number of samples in each group in Table 3). The original scanned and binarized images of the nine genera of leaves are shown in Fig. s1. The contours are traced using the Moore-Neighbor method^32^ and then smoothed with the MATLAB cubic spline interpolation (see Fig. s2). All leaf profiles of their original sizes are analyzed. The arc lengths of the profiles range from 1141.5 units to 8433.1 units, the areas of the leaves range from $6.1671\times {10}^{4}$ units^2^ to $1.4615\times {10}^{6}$ units^2^.

Applying the relative angle method, a number of apices are recognized on each leaf contour. These apices are the primary segmentation points that determine the boundaries of sub-profiles on leaf contours. Note that the shapes of leaves from different groups vary significantly, therefore the point interval and angle threshold used for locating apices varies from group to group. For some groups, the numbers of apices identified on different samples may be different too. Table 4 shows the parameters used for identifying apices as well as the minimum and maximum numbers of apices identified on samples for each group.

**Table 4:** Parameters used for identifying apices on leaf contours and the number of apices identified for each group.

Group	Profile index	Number of samples $n$	Point interval $k$	Angle threshold $T$ (degree)	Number of apices	
					Min	Max
Cherry	1–18	18	300	80	1	1
Dogwood	19–32	14	300	80	1	1
Gum	33–48	16	500	60	9	10
Hickory	49–64	16	300	60	1	1
Mulberry	65–81	17	475	79	1	1
Red maple	82–100	19	400	62.5	5	5
Red oak	101–111	11	325	60	22	34
Sugar maple	112–129	18	110	40	19	29
White oak	130–145	16	275	30	13	20

In order to maintain homology, supplementary segmentation points are added to divide all sample profiles into the same number of portions. There is no need to add more segmentation points on the profile that contains the most number of apices (red oak, $j=101$), therefore the total number of segmentation points on each profile is determined to be 34, dividing each profile into 35 portions. In order to reduce the matching error, supplementary segmentation points are distributed as evenly as possible in sub-profiles formed by the primary segmentation points (original apices) using a method developed based on a genetic algorithm (GA). In this problem, the locations of the supplemented segmentation points on the ${j}{\text{th}}$ profile are determined through the fitness function determined as follows

11 $${F}_{j}=\sum_{e=1}^{q}{\left({k}_{j}^{e+1}-{k}_{j}^{e}-\frac{{N}_{j}-1}{q}\right)}^{2}.$$

**Figure 9:**
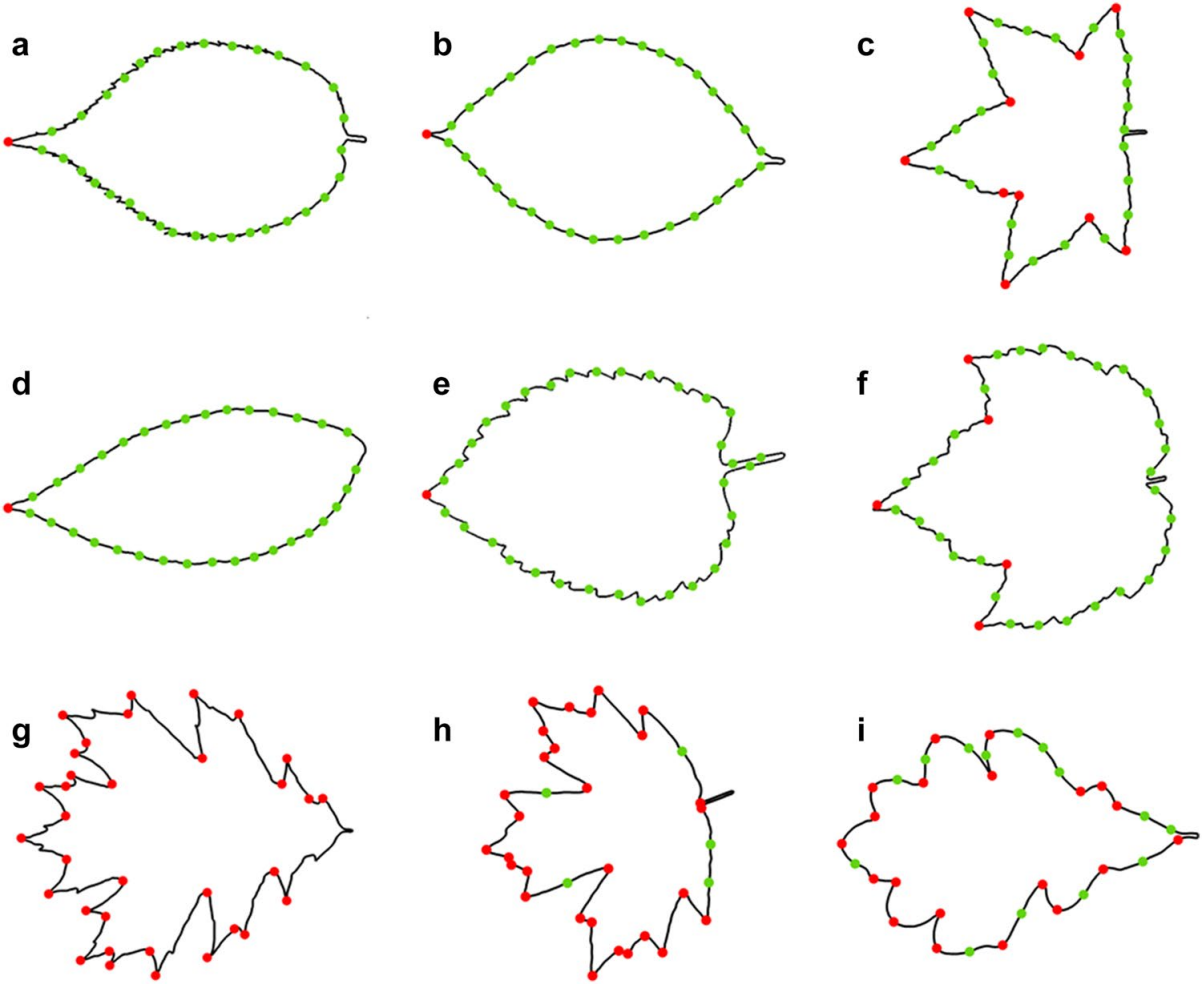
The original apices (red circles) and supplementary segmentation points (green circles) on leaf contours. (**a**) Cherry, (**b**) Dogwood, (**c**) Gum, (**d**) Hickory, (**e**) Mulberry, (**f**) Red maple, (**g**) Red oak, (**h**) Sugar maple, (**i**) White oak. For each group, the sample that contains the most original apices is presented.

In Eq. (11), the number of pieces contained in the ${j}{\text{th}}$ profile $({N}_{j}-1)$ divided by the number of portions $q$ yields the average number of pieces in each portion. $({k}_{j}^{e+1}-{k}_{j}^{e})$ is the number of pieces contained in the ${e}{\text{th}}$ portion confined by the ${e}{\text{th}}$ and the ${(e+1)}{\text{th}}$ segmentation points on the ${j}{\text{th}}$ profile. After encoding the locations of all segmentation points in the GA and several rounds of optimization based on a certain scale of crossover and mutation, the set of supplementary segmentation points that minimizes the fitness function, Eq. (11), is determined. The original apices (red circles) and supplementary segmentation points (green circles) distributed on samples from different groups are shown in Fig. 9. In this example, each profile is finally divided into 35 portions.

**Figure 10:**
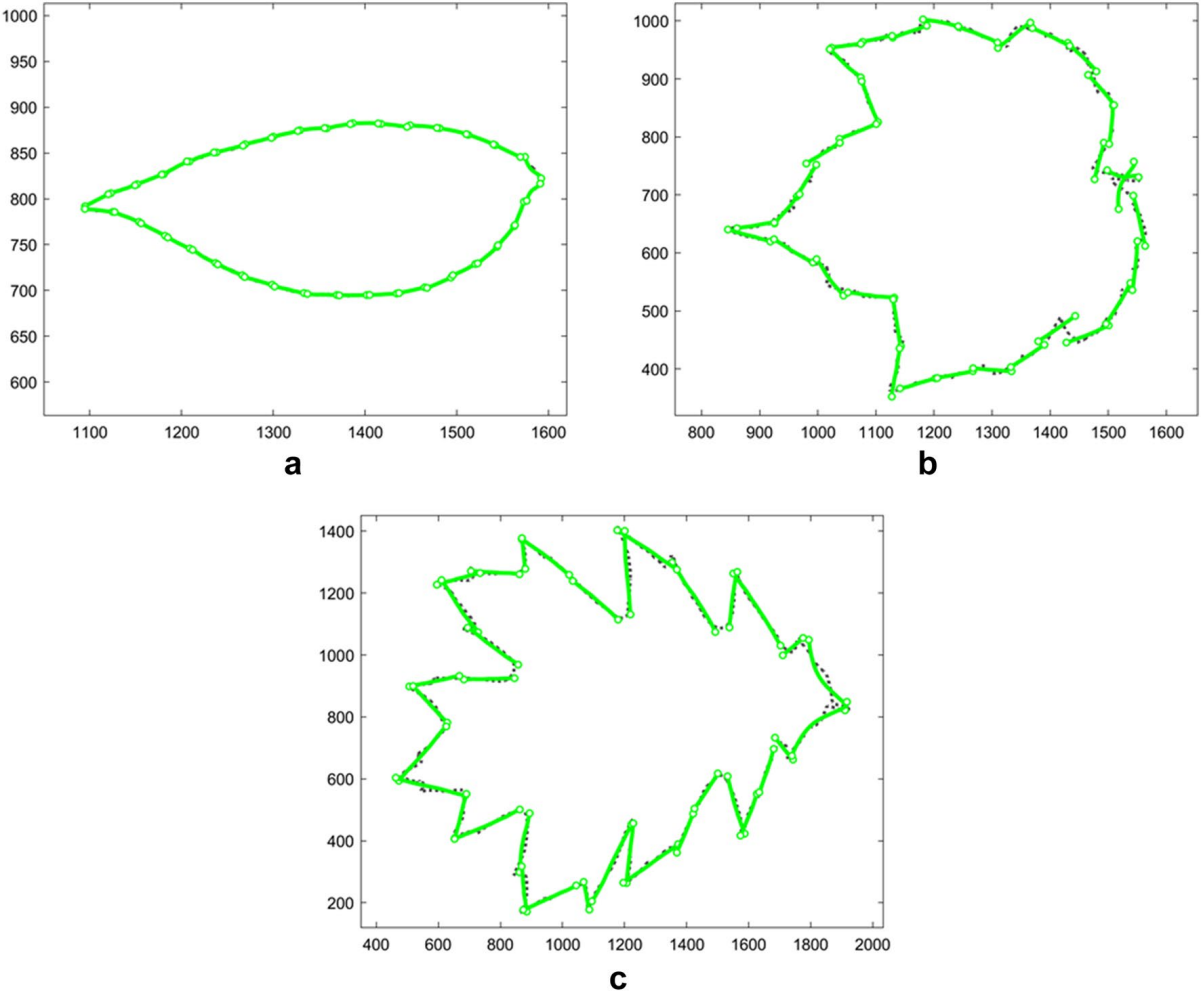
The fitting results of 145 leaves. (**a**) The best match (the 62nd profile—hickory, ${\tilde{E }}_{62}=0.8748$); (**b**) The average match (the 88th profile—red maple, ${\tilde{E }}_{88}=4.0866$); **c** The worst match (the 110th profile—red oak, ${\tilde{E }}_{107}=9.7866$).

The length of each portion varies among profiles, thus M-segments are not applicable. In addition, some portions still contain local burrs and sharp corners, which would not be matched well by C-segments. Therefore, each portion is matched by a G-segment, and the segment vector contains 35 G-segments. The maximum and mean error of 145 leaf profiles are ${E}_{\text{max}}=60.6063$ and $\overline{E }=8.7062$ units, respectively. Figure 10 shows the best, the average, and the worst matching results of the leaves according to ${\tilde{E }}_{j}$. More matches of nine genera of leaves are illustrated in Fig. s3. The result show that given the distribution of apices (primary segmentation points that determine sub-profiles), the GA strategy can automatically determine the distribution of supplementary segmentation points along a profile. With the segmentation points generated from this process, the shape-changing chain matches the leaf contours with small error compared to the random segmentation in the previous study.

**Figure 11:**
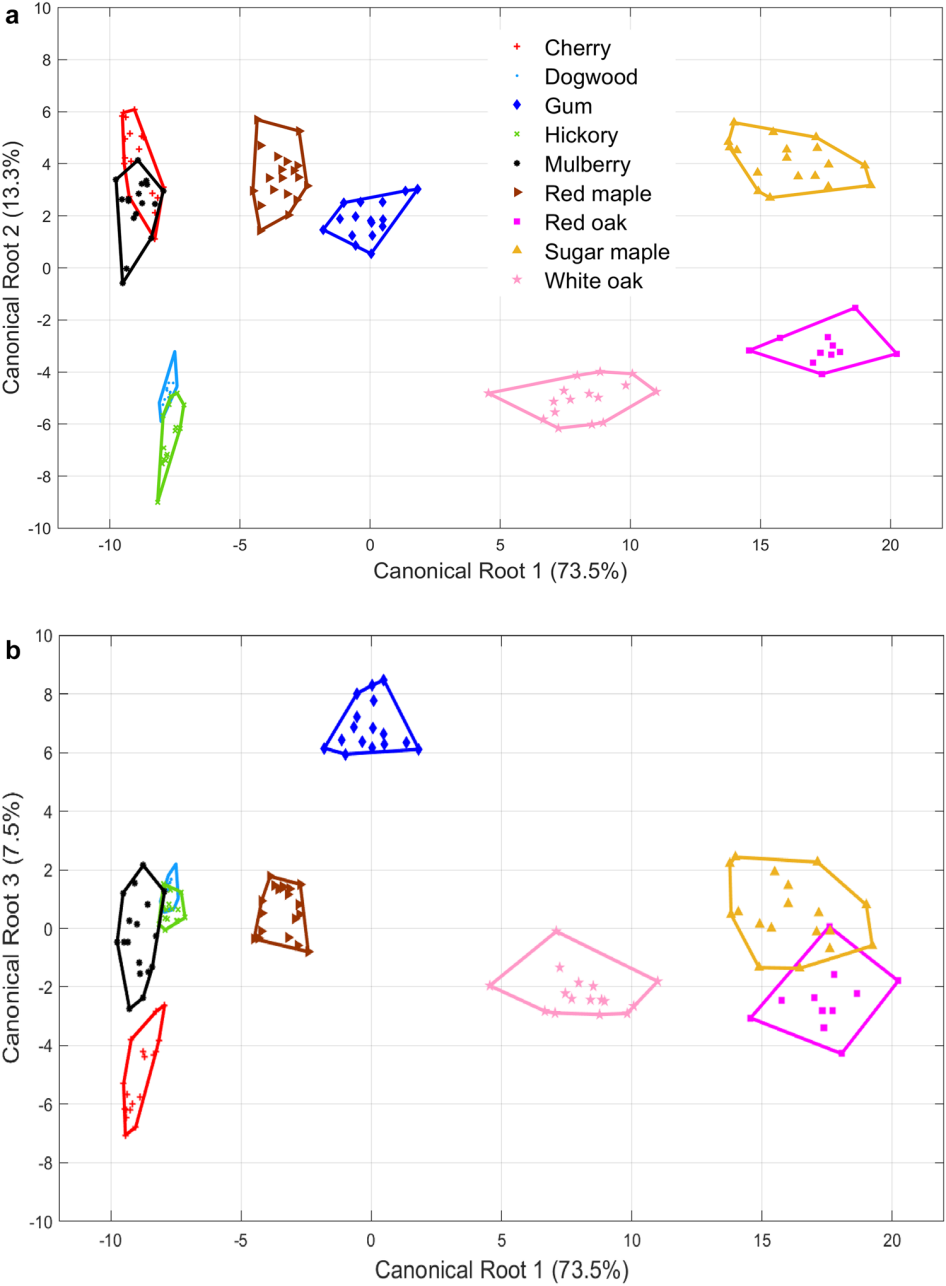
The 2D Canonical plots of nine genus of leaves based on 22 variables.

**Figure 12:**
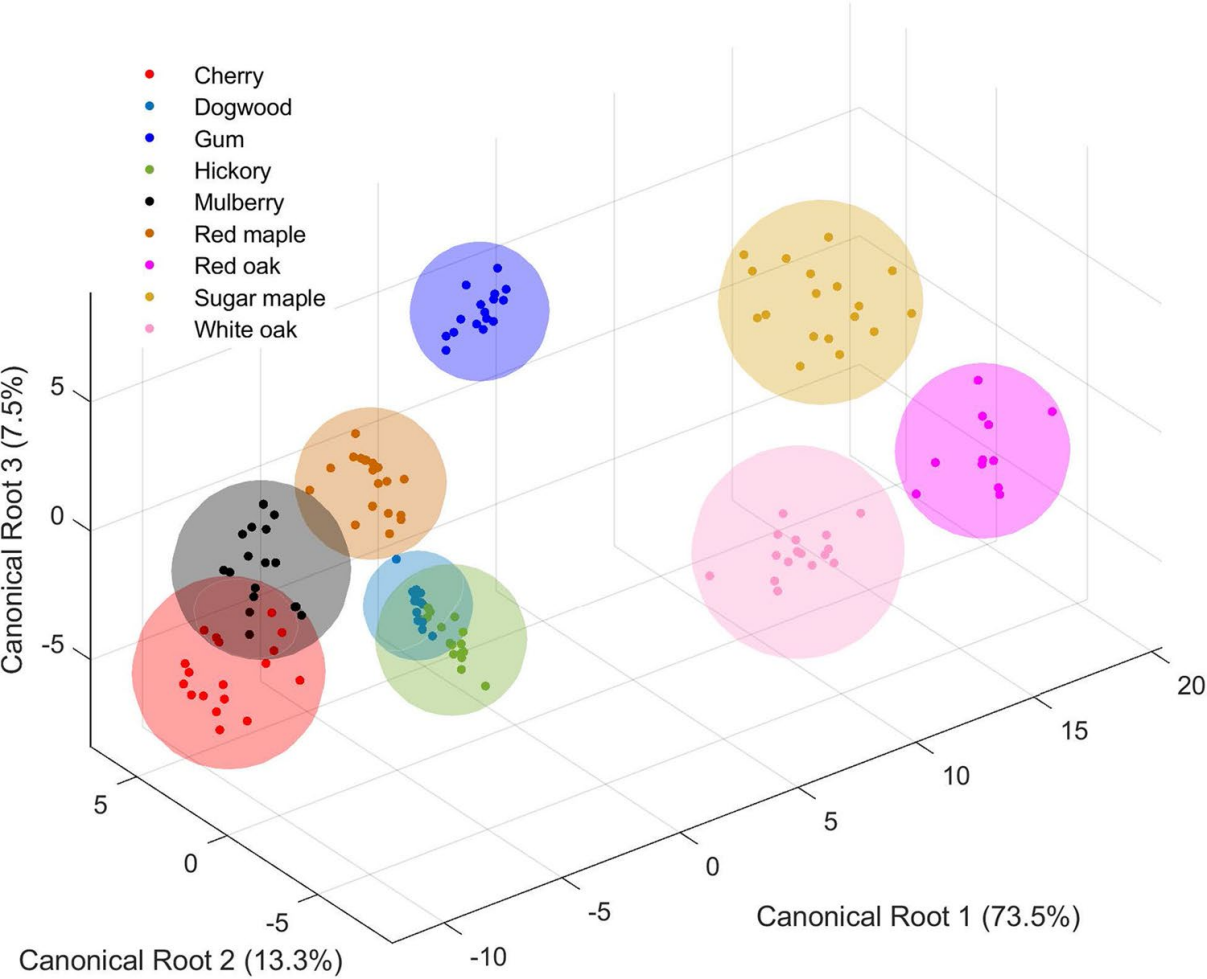
The 3D Canonical plot of nine genus of leaves based on 22 variables.

For classification analysis, 34 orientation differences between neighboring segments are calculated using Eq. (10). Three more variables are employed: The number of primary segmentation points, the number of burrs (detected using the relative angle method with $k=50$ and $T=30^\circ$), and the arc length of each profile. This sums up to a total of 37 variables. A stepwise DA is performed to classify the 145 leaf samples, and 22 out of the 37 variables are selected for analysis. The variances of the first three canonical functions are 73.5%, 13.3%, and 7.5%, which add up to 94.3% in total. Figures 11 and 12 illustrate the 2D and 3D canonical plots of the nine genus of leaves based on the first three canonical components. The plots show that gum, red maple, and white oak are distinctively separated from other groups. Cherry and mulberry are partially overlapped in the directions of canonical Roots 1 and 2 for their similar overall shapes and serrated edges. There is also an overlap between dogwood and hickory in the directions of canonical Roots 1 and 3 for their similar shapes and smooth edges. The prediction accuracy is 98.6%, and the leave-one-out cross-validation is 97.9%. Only two samples of cherry are misidentified as mulberry, and one sample of hickory is discriminated as dogwood. The DA results reveal that the shape-changing method is capable of fitting a large number of profiles that have complicated shapes and different sizes, as well as generating useful variables for statistical analysis. The leave-one-out cross-validation accuracy suggests that this method is also effective with fewer variables. In addition, the shape-changing chain method enables direct observation and comparison of variables that have physical meanings, such as the relative angles between segments.

### 3D cranial suture curves

**Figure 13:**
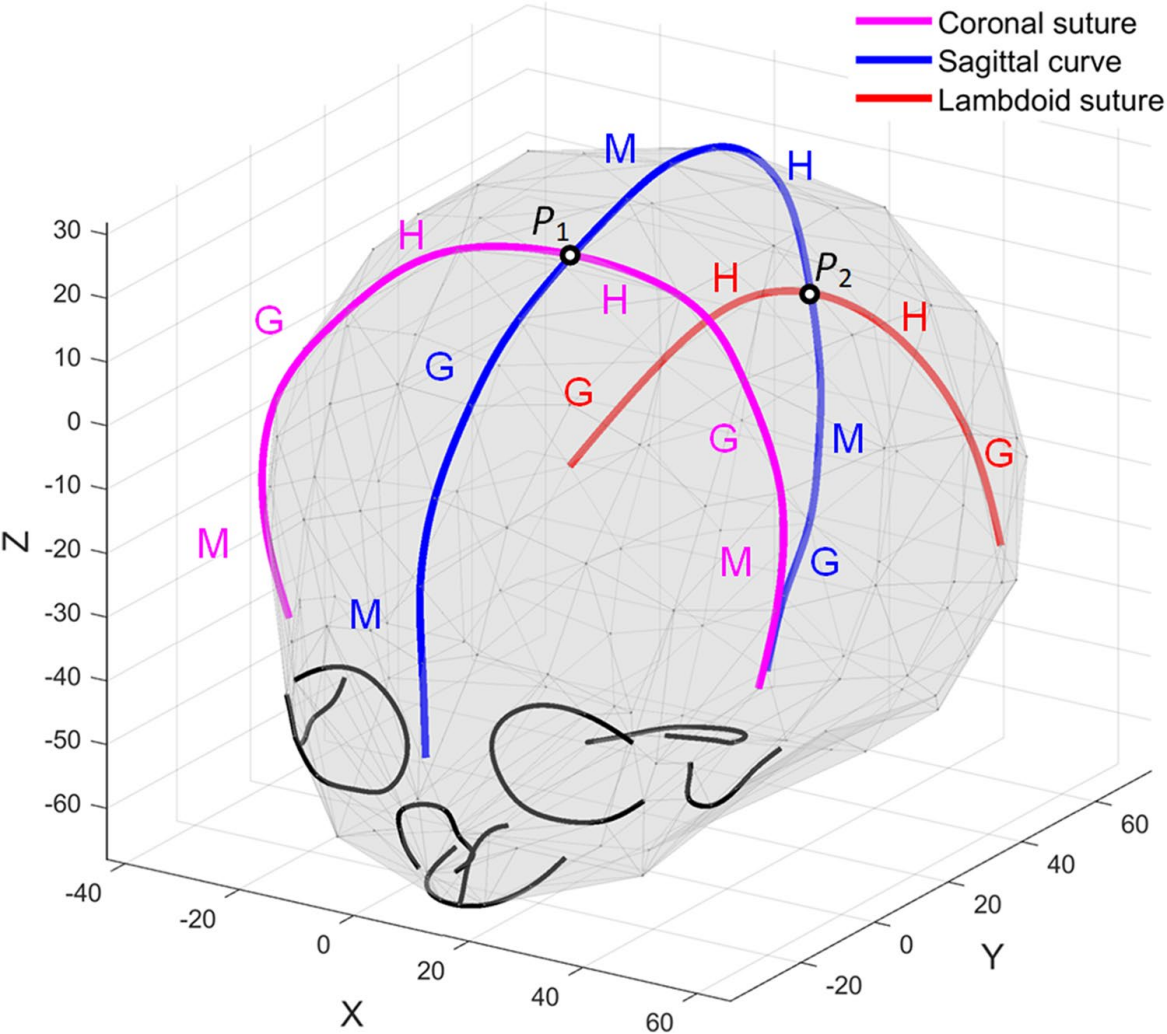
The location of the coronal suture (magenta), sagittal curve (blue) and the lambdoid suture (red) on a human infant skull. The intersection points between sutures, *P*_1_ and *P*_2_, divide the sagittal curve into three sub-profiles and the lambdoid suture into two sub-profiles.

The shape-changing chain method is now applied to 3D suture curves on human infants’ skulls from a study of coronal synostosis^18,19^. The dataset contains 63 samples categorized into 4 groups, including left unicoronal synostosis (LUCS, $n=8$), right unicoronal synostosis (RUCS, $n=19$), bicoronal synostosis (BCS, $n=16$), and unaffected cases ($n=20$). The original data of each sample consist of 209 anatomical landmarks and curve semilandmarks located on the skull surface, especially along some anatomical lines as sutures. In this work, three curves that characterize the skull deformation are selected for analysis: the coronal suture curve, the lambdoid suture curve, and the sagittal curve which is comprised of anatomical landmarks and curve semilandmarks located on the metopic suture, the sagittal suture, and the mid-line on the occipital bone. Figure 13 shows the three suture curves on a skull surface.

**Figure 14:**
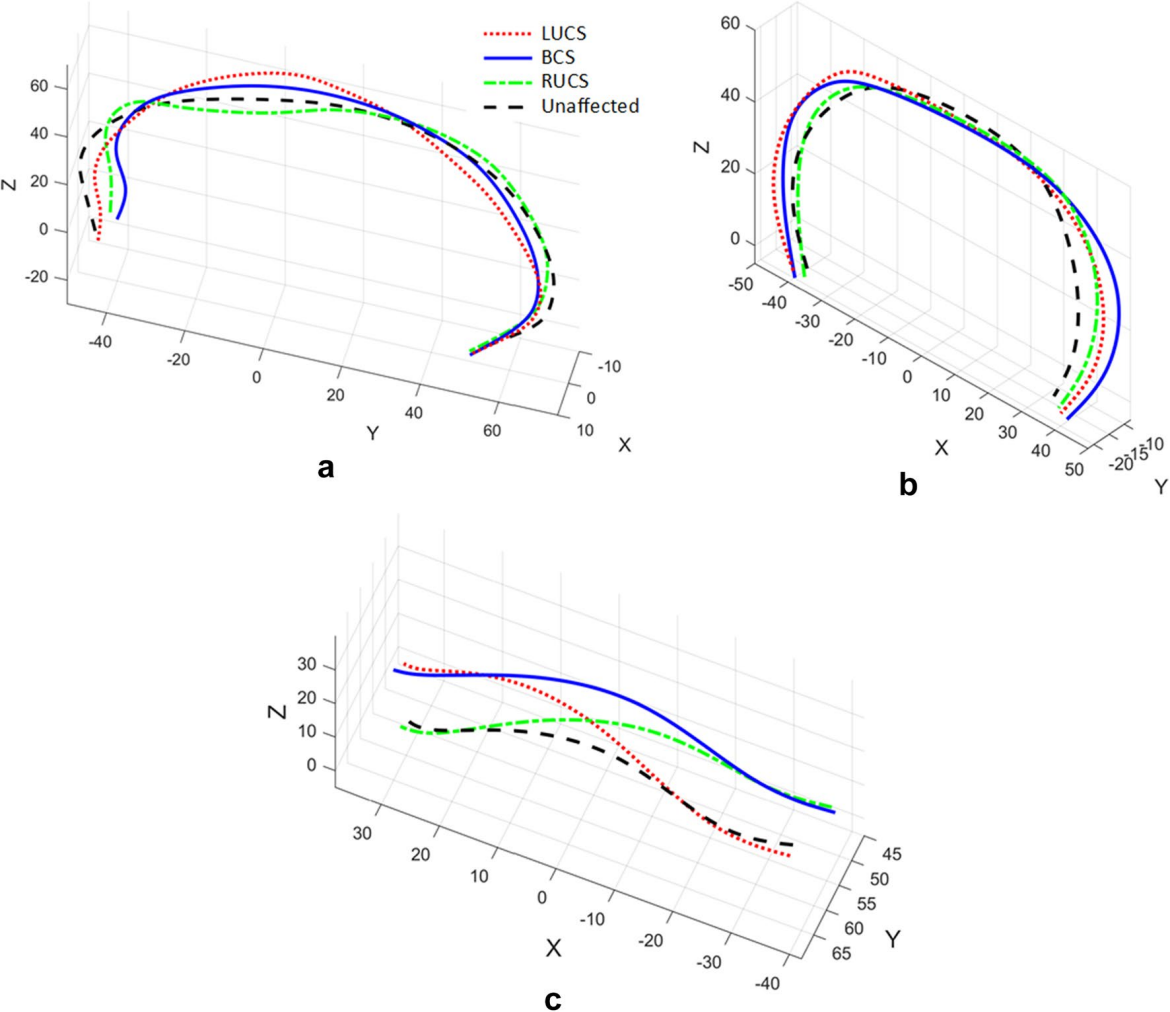
Mean shapes of (**a**) the sagittal curves, (**b**) the coronal suture, and (**c**) the lambdoid sutures of four groups: LUCS (red dotted line), BCS (blue solid line), RUCS (green dotted dashed line), and unaffected cases (black dashed line). Notice the symmetry of the suture curves about the skull symmetry plane (X = 0).

BCS occurs when the coronal sutures on both sides of the skull fuse prematurely, causing the overall head shape to become broad and short. In this case, the relative location of the lambdoid suture on the skull will move forward compared to the unaffected cases, but its shape is not affected as obviously as the coronal suture or the sagittal curve which are directly affected by coronal synostosis. In order to investigate the relative location and orientation in addition to the shape of the suture curves, a standard Procrustes superimposition is performed on the original data so that all skulls represented by the 209 landmarks and semilandmarks are scaled to the same size and aligned. Figure 14 illustrates the mean shapes of the sagittal curves and the lambdoid sutures of each group. It can be observed that for BCS cases, the sagittal curve is shorter in the anterior–posterior direction, the coronal suture becomes wider in the left–right direction, and the lambdoid suture is longer and positioned relatively forward. These differences are in accordance with the overall wider and shorter BCS skull shape. As for LUCS and RUCS cases, all three curves display a symmetrical shape deformation or orientation change about the skull symmetry plane (X = 0).

The anatomical landmarks *P*_1_ and *P*_2_ (Fig. 13) which are the intersection points between the sutures, are selected as the primary segmentation points. Thus, the sagittal curve, the coronal suture, and the lambdoid suture are divided into three, two, and two sub-profiles, respectively. Since the coronal suture and the lambdoid suture grow symmetrically about the skull symmetry plane, their segment type vectors for two sub-profiles should be symmetric, respectively. In addition, each segment type vector should contain a G- or H-segment to characterize the growth. The segment type vectors of the three curves are designated as:

- Sagittal curve: $$\left[\begin{array}{cc}{\text{M}}& {\text{G}}\end{array}\right]$$, $$\left[\begin{array}{cc}{\text{M}}& {\text{H}}\end{array}\right]$$, $$\left[\begin{array}{cc}{\text{M}}& {\text{G}}\end{array}\right]$$;
- Coronal suture:$$\left[\begin{array}{ccc}{\text{M}}& {\text{G}}& {\text{H}}\end{array}\right]$$, $$\left[\begin{array}{ccc}{\text{H}}& {\text{G}}& {\text{M}}\end{array}\right]$$;
- Lambdoid suture: $$\left[\begin{array}{cc}{\text{G}}& {\text{H}}\end{array}\right]$$, $$\left[\begin{array}{cc}{\text{H}}& {\text{G}}\end{array}\right]$$.

**Figure 15:**
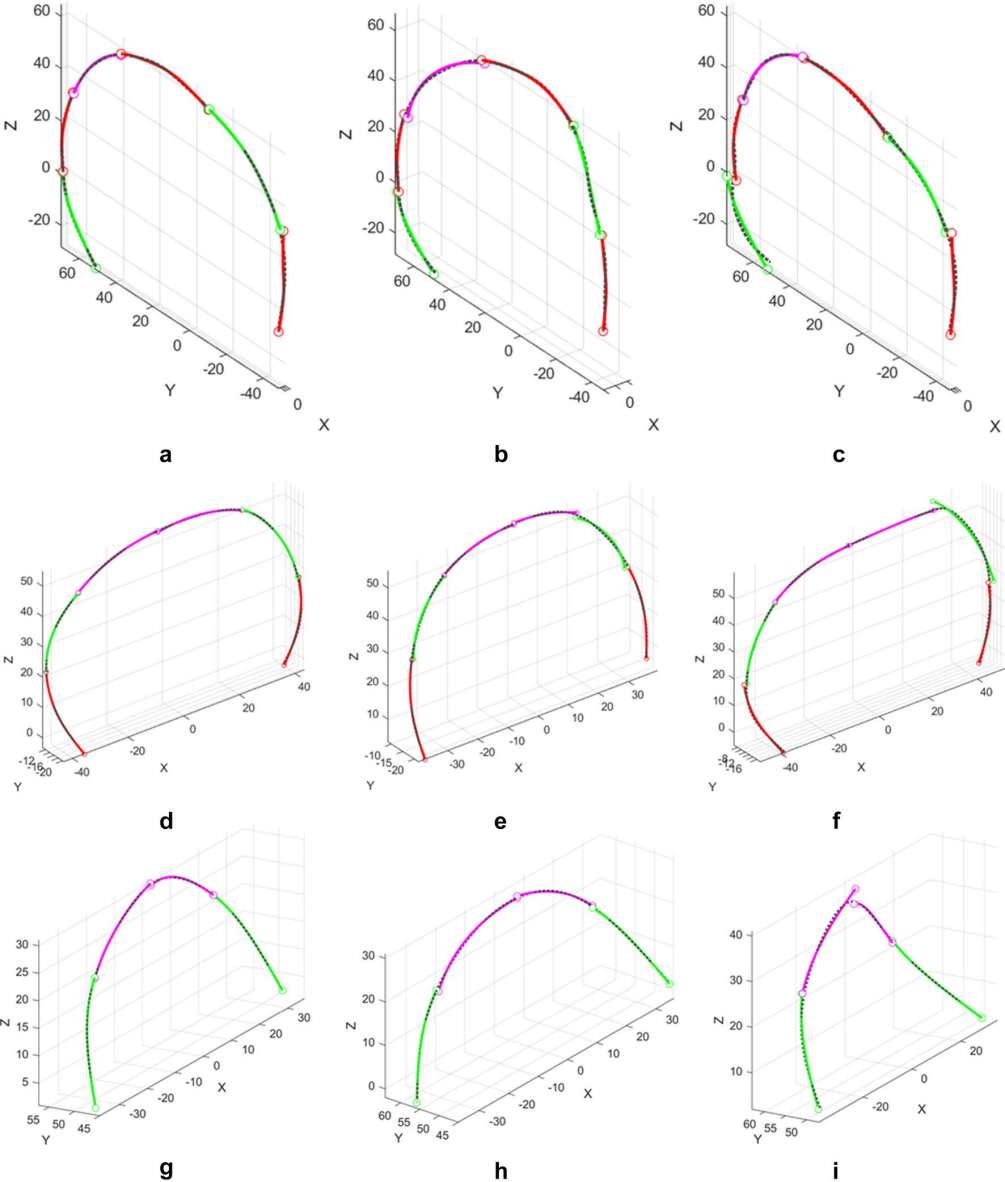
The fitting results of (**a–c**) sagittal curves, (**d–f**) coronal sutures, and (**g–i**) lambdoid sutures from 63 samples. The left column (**a**, **d**, **g**) is the best match of each group, the middle column (**b**, **e**, **h**) is the average match of each group, and the right column (**c**, **f**, **i**) is the worst match of each group. (**a**) ${\overline{E} }_{s-52}=0.5122$ (unaffected); (**b**) ${\overline{E} }_{s-19}=0.8681$ (BCS); (**c**) ${\overline{E} }_{s-46}=1.6055$ (unaffected); (**d**) ${\overline{E} }_{c-12}=0.2507$ (BCS); (**e**) ${\overline{E} }_{c-54}=0.5082$ (unaffected); (**f**) ${\overline{E} }_{c-42}=0.9636$ (RUCS); **g** ${\overline{E} }_{l-29}=0.1225$ (BCS); (**h**) ${\overline{E} }_{l-37}=0.3687$ (BCS); (**i**) ${\overline{E} }_{l-28}=1.0997$ (RUCS).

In spatial cases, the orientation of each segment is given by 3 parameters, and each G- or H-segment is characterized by an additional length parameter. Therefore, this matching scheme generates 21, 22, and 16 parameters to describe the shape variances for the sagittal curves, the coronal suture curves, and the lambdoid suture curves, respectively. Note that the suture curves are relatively smooth, thus the average value of the maximum error on all segments ${\overline{E} }_{j}$ is very significant of the matching error of the chain at the ${j}{\text{th}}$ profile. Therefore, this parameter is chosen to assess the error in this application. Figure 15 shows the best, the average, and the worst matches of the sagittal curves, the coronal sutures, and lambdoid sutures. The overall mean error ($\overline{E }$) of the sagittal curves, the coronal sutures, and the lambdoid sutures are 0.8728, 0.5060, and 0.3666 units, respectively.

In order to represent the orientation of the spatial chain, the ${e}{\text{th}}$ segment at the ${j}{\text{th}}$ profile is characterized by a unit vector that points from the starting point to the endpoint of the segment as

12 $${\mathbf{u}}_{j}^{e}=\frac{{\overline{\mathbf{z}} }_{{j}_{{m}_{j}^{e}+1}}^{e}-{\overline{\mathbf{z}} }_{{j}_{1}}^{e}}{\Vert {\overline{\mathbf{z}} }_{{j}_{{m}_{j}^{e}+1}}^{e}-{\overline{\mathbf{z}} }_{{j}_{1}}^{e}\Vert }.$$

**Figure 16:**
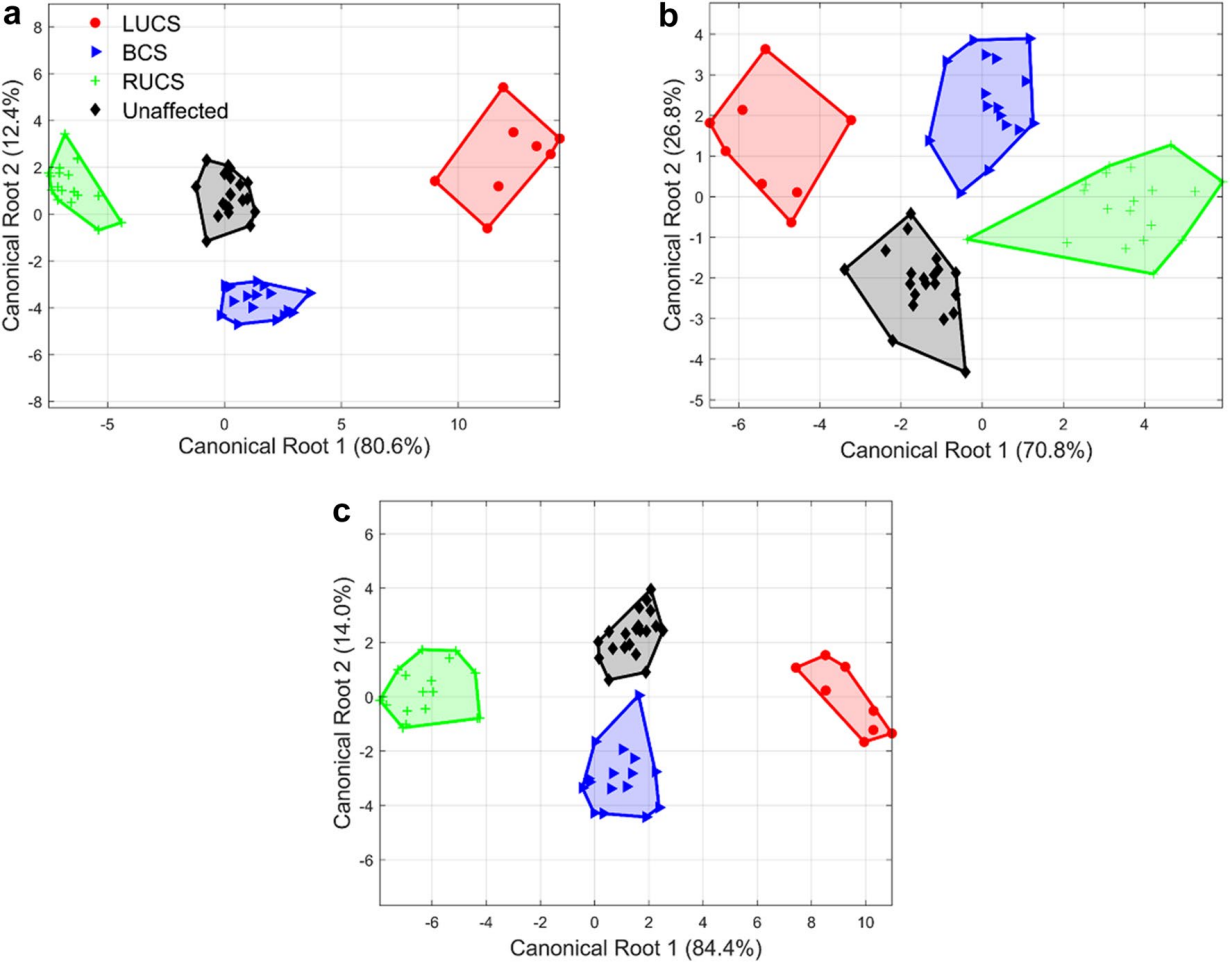
The 2D canonical plots of the suture curves selected from 63 skull samples. (**a**) The sagittal curves based on 6 variables. (**b**) The coronal sutures based on 7 variables. (**c**) The lambdoid sutures based on 8 variables.

Since each vector ${\mathbf{u}}_{j}^{e}$ contains three Cartesian coordinates, each sagittal curve, coronal suture, and lambdoid suture is thus characterized by 18, 18, and 12 variables, respectively. For lambdoid sutures, its relative location which is characterized by the coordinates of point *P*_2_ is also analyzed. Therefore, the total number of variables analyzed for a lambdoid suture is 15. This is much fewer than the 209 landmarks and semilandmarks analyzed in the work of Heuzé et al.^19^. Stepwise DA is conducted with the variables above, and LOOCV is performed to verify the stability of the linear model. In stepwise DA, 6, 7, and 8 variables are selected to be analyzed for sagittal curves, coronal sutures, and lambdoid sutures, respectively. Figure 16 illustrates the canonical plots of the three set of curves. As shown in Fig. 16, all three set of suture curves display strong separation among four classes on the 2D canonical plots. Besides, the LUCS and RUCS curves are distributed in the opposite directions from the BCS and unaffected ones along the first canonical component, while the BCS and unaffected curves differ in the direction of the second canonical component. These plots confirm the symmetrical shape deformation of the suture curves of LUCS and RUCS cases, and the changes in the lengths and relative locations of the suture curves of BCS as observed in Fig. 14.

The original DA prediction accuracy and cross-validated accuracy are both 100% for the sagittal curves, which indicates that the shape difference of the sagittal curves can efficiently distinguish specific diagnosis of coronal synostosis. The original DA prediction accuracy and cross-validated accuracy for the coronal sutures are 98.4% and 96.8%, respectively. There are two cases (BCS and RUCS) misclassified as unaffected case. As for the lambdoid sutures, the original DA prediction accuracy and cross-validated accuracy are both 98.4%. The only misclassified case in both predictions is that one BCS lambdoid suture is categorized as unaffected. This suggests that the coronal suture and the lambdoid suture is subjected to both shape deformation and location transformation due to coronal synostosis.

These matching and classification results show that the shape-changing chain is efficient in fitting and analyzing 3D curves with a very moderate number of variables compared to other parametric methods. For example, Zhou et al. employed discrete cosine transform (DCT) to analyze the same three sets of suture curves^33^. In their work, 12, 6, and 6 harmonics are employed to fit the sagittal curves, the coronal suture curves, and the lambdoid suture curves, resulting in 36, 18, and 18 coefficients to be analyzed. Table 5 shows a comparison of the variables used to match the curves and perform statistical analysis with the two methods. A comparison of the classification accuracies of the two methods is not provided because Zhou et al. employed between-group principal component analysis (bgPCA) while the presented work uses stepwise DA. Note that the variables obtained in DCT are mathematical coefficients which are hard to interpret, while the variables in the shape-changing chain method represent the orientations, lengths, or locations of segments, providing direct information of the variance of the curve shapes.

**Table 5:** Numbers of variables used in the shape-changing chain method and in DCT^33^ for fitting and analyzing suture curves.

Profile	Method	Variables in fitting curves	Variables to be analyzed
Sagittal curve	Shape-changing chain method	21	18
	DCT	36	36
Coronal suture curve	Shape-changing chain method	22	18
	DCT	18	18
Lambdoid suture curve	Shape-changing chain method	16	15
	DCT	18	18

## Discussion

In this paper, a method to fit and compare the forms of a group of biological curves (profiles) via a single shape-changing chain is presented. The profiles to be analyzed can be 2D or 3D, open or closed, smooth or serrated. The shape-changing chain is composed of three types of segments, M-segments, H-segments (or C-segments for 2D profiles), and G-segments. The choice of the segment types is directly related to the application: it may be based on some geometrical considerations (generally speaking, H-/C-segments are more suitable for regions with constant curvature and torsion, while G-segments are more suitable for regions that have similar shapes), on some extra knowledge or hypothesis (as for example, the growth mechanism of the mandible ), or on some experiments where we test different segment types and choose the scheme that yields the best classification result and lower matching error. The segmentation strategy considers both biological and geometrical aspects to establish correspondence of segments among different profiles. Besides anatomical landmarks, high-curvature points are captured using the relative angle method and also utilized as segmentation points. Note that other useful information should be considered likewise for more accurate approximation, such as the energy of the system (see for example^2^ and^34^). The segment matching result is optimized via two approaches. The first one regenerates segment lengths for profiles that are badly matched, while the matching scheme for the rest of the profiles remain unchanged. The second approach modifies segment lengths defined by the segment matrix according to the error matrix so that segments with higher matching error are shortened while segments with lower matching error are elongated. Both optimization approaches are conducted in an iterative fashion. After the fitting chain is obtained, DA is performed based on the kinematic parameters of the chain and other variables from the fitting results, such as the orientation of segments, the orientation differences between neighboring segments, the number of global apices and local burrs captured on each profile.

It should be emphasized that the shape-changing chain method is applicable to general problems, but each new problem requires some tinkering to tailor the tool for the data set. The entire process can be divided into three parts: choosing a segment type vector, generating a matching result, and analyzing the matching data. During the process of choosing a segment type vector, a designer who is familiar with the evolution/growth/taxonomy/etc. can provide educated suggestions. The move from segment type vector to matching segments is quite methodical and generally applicable. The process of analyzing the matching data also requires user interaction to select appropriate data. Therefore, for a specific problem, there is work for designer to do in both choosing the segment type and selecting meaningful matching data, but a stated and clear plan of attack has been developed in this paper.

Three examples are presented in this paper, including 2D open profiles of fossil hominid mandibles ($n=94$), 2D closed profiles of leaf contours ($n=145$), and 3D open profiles of suture curves on human infant skulls ($n=63$). The fitting results and the discriminant results of the three examples demonstrate that the shape-changing chain method is applicable and reliable in morphometric applications. Moreover, the shape-changing chain method has several advantages compared to current mainstream morphometric methods.

First, the shape-changing chain method can be applied in a wider range of problems. For the shape-changing chain method, the profiles to be analyzed can be 2D or 3D, open or closed, smooth or serrated, and vary in size or arc length. As for GM, most data require GPA to remove the size factor. Elliptical Fourier analysis, the most common outline-based GM method, has been used to trace 2D open outlines in some cases, but was originally developed for 2D closed profiles. In addition, this method has trouble dealing with outlines having a symmetrical shape.

Second, the shape-changing chain method admits flexibility in balancing between biological homology and human intervention in the curve-fitting process. When it comes to the morphometric analysis of curves or surfaces, homology should not be ignored. GM methods such as the semilandmarks method, EFA, or the elastic theory are based on landmarks which are manually located using anatomy knowledge and on semilandmarks which are interpolated between landmarks. As for smooth curves where prominent anatomical landmarks are lacking, the position of anatomical landmarks may be difficult to estimate precisely and the interpolated semilandmarks may vary from one sample to another. The resulting mathematical homology (correspondence of semilandmarks between different samples) may be not consistent with the biological homology. In the case of the shape-changing chain, biological knowledge can be used to infer constraints for segmenting smooth curves (e.g. Enlow’s growth pattern for the mandible^28^). The global constraints based on biological information will automatically result, by mathematical optimization, in segments which are the equivalent of semilandmarks for GM.

Third, the shape-changing chain can obtain satisfying morphometric analysis results from fewer variables than GM. Landmark-based GM often requires hundreds of landmarks and semi-landmarks, which leads to data redundancy and thus brings difficulty in the statistical analysis. In outline-based GM, such as the EFA approach, the number of variables is proportional to the specified number of harmonics which must be quite high in most cases to reach a good accuracy.

Fourth, the variables obtained from the shape-changing chain reflect the nature of the deformation of outlines, while the variables obtained from the fitting results of GM methods are merely mathematical coefficients. For instance, the difference between the orientations of two neighboring segments reflects the bending between two portions on a curve, and the number of pieces contained in a G-, H-, or C-segment at different profiles reflects the difference in the length of corresponding portions on different samples.

## Conclusion

The shape-changing chain method extended from mechanical synthesis methodology has been presented to be applicable to a wide range of morphometric problems involving 2D or 3D, open or closed outlines. This method takes both biological homology and mathematical correspondence into account during the curve-fitting process. An optimization method for segmentation is presented for problems involving a large number of profiles. A moderate number of parameters obtained from the fitting results can be used for statistical analysis, and show physical interpretations of the differences and similarities among profiles. In conclusion, the shape-changing chain method can be a versatile tool for morphometric analysis and bring a new perspective to the study of morphology. Future work includes improving the method for smooth curves without prominent landmarks, such as the cochlear and the corpus callosum profiles, and automated identification of segmentation.

## Supplementary Information

Supplementary Information.
