# Supplementary material for: Shape-changing chains for morphometric analysis of 2D and 3D, open or closed outlines
Source: Sci Rep. 2021 Nov 2;11:21479. doi: 10.1038/s41598-021-00911-5 (PMC8563779; doi:10.1038/s41598-021-00911-5)
Supplement: Supplementary file 1 — Supplementary Information. [file 41598_2021_911_MOESM1_ESM.docx]

**Shape-changing chains for morphometric analysis of 2D and 3D, open or closed outlines**

Bingjue Li*, Shengmin Zhou, Andrew Peter Murray, Gérard Subsol

**Correspondence: libj@seu.edu.cn*

**Supplementary materials**

|  |  |  |
| --- | --- | --- |
| **a** Cherry | **b** Dogwood | **c** Gum |
|  |  |  |
| **d** Hickory | **e** Mulberry | **f** Red maple |
|  |  |  |
| **g** Red oak | **h** Sugar maple | **i** White oak |
| **Figure s1.** Examples of the scanned and binarized images of nine genera of leaf samples. | | |


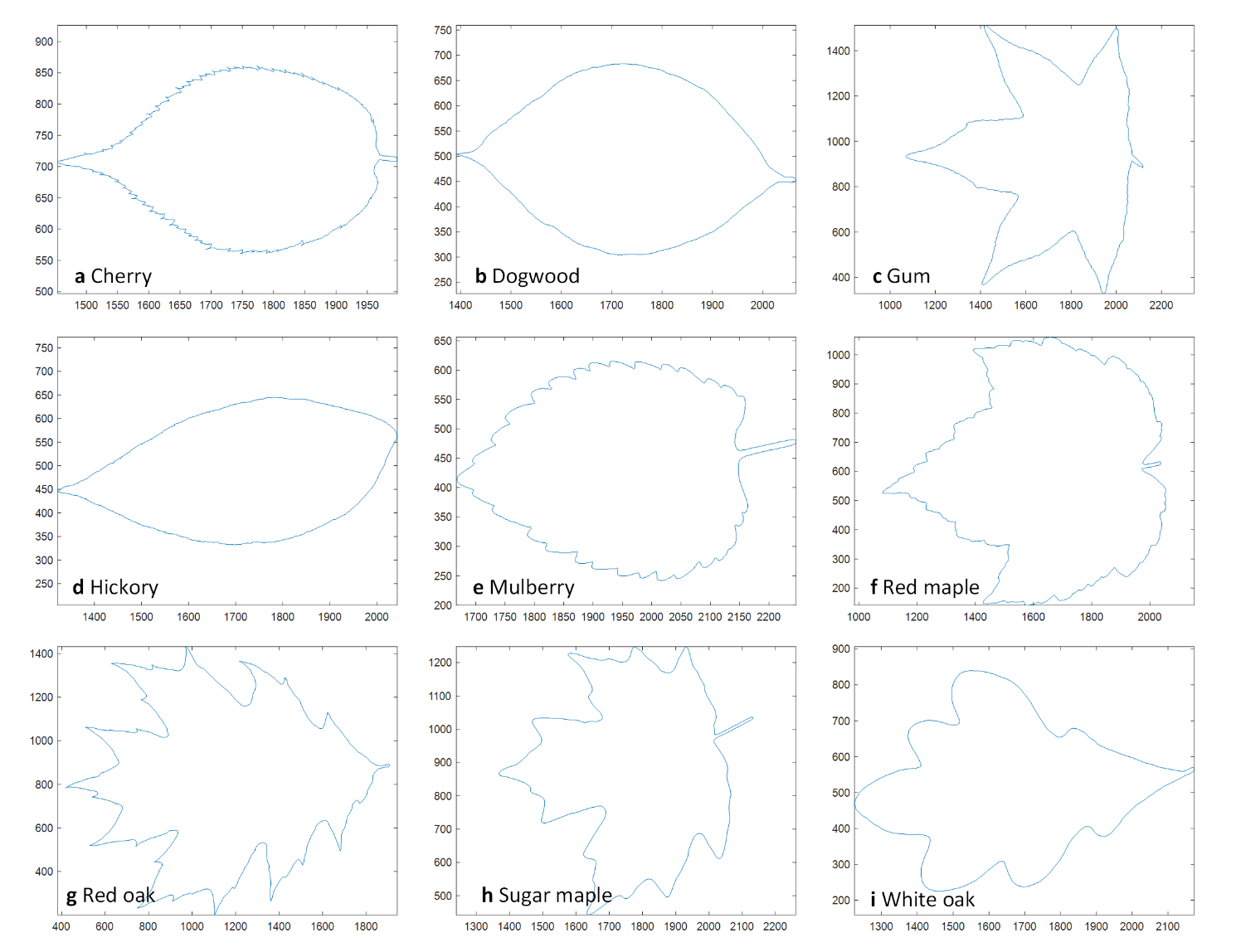


**Figure s2.** Examples of the leaf contours of nine genera traced using the Moore-Neighbor method.


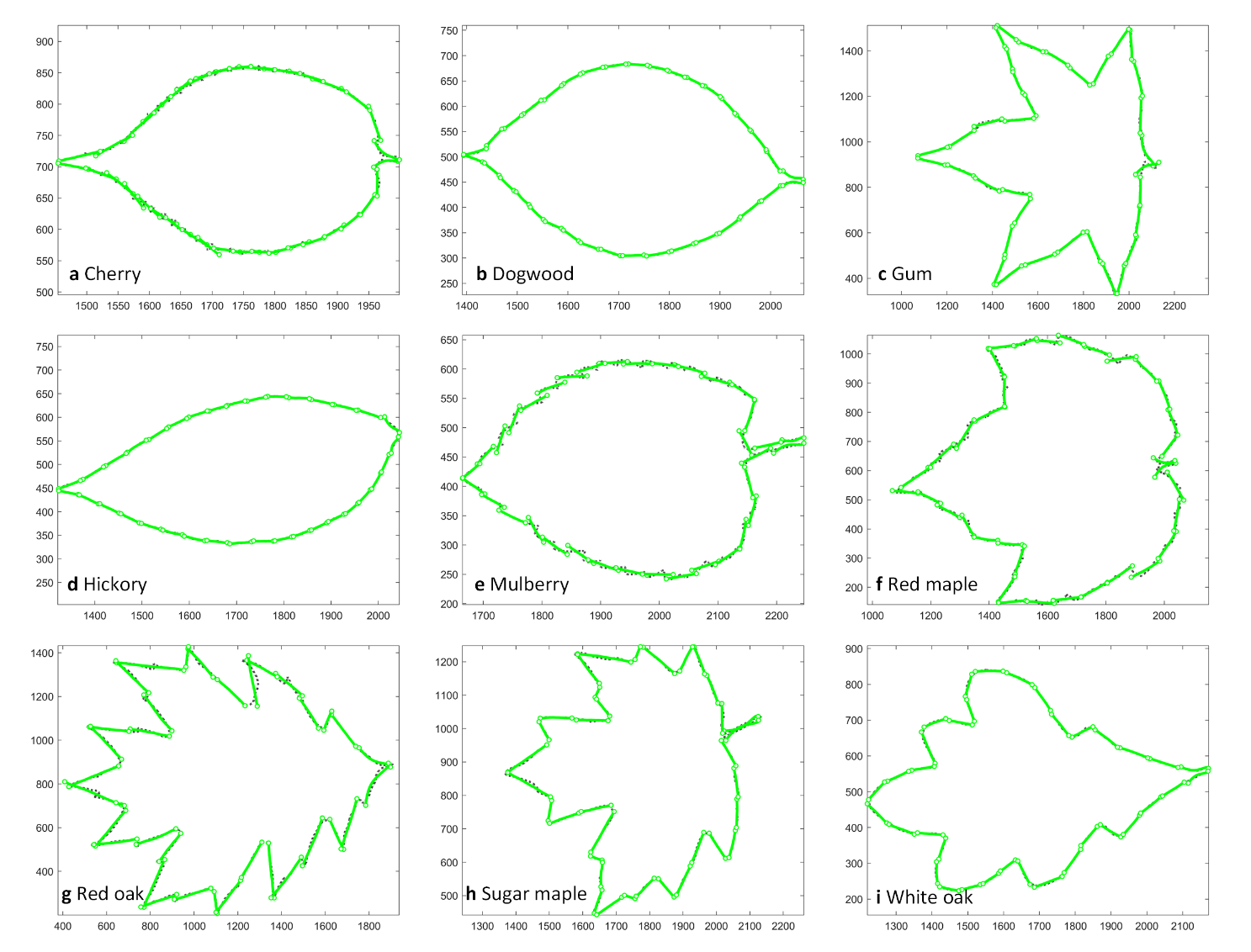


**Figure s3.** Example matches of nine genera of leaves.
